# Systematic identification of novel cancer genes through analysis of deep shRNA perturbation screens

**DOI:** 10.1093/nar/gkab627

**Published:** 2021-07-27

**Authors:** Hesam Montazeri, Mairene Coto-Llerena, Gaia Bianco, Ehsan Zangene, Stephanie Taha-Mehlitz, Viola Paradiso, Sumana Srivatsa, Antoine de Weck, Guglielmo Roma, Manuela Lanzafame, Martin Bolli, Niko Beerenwinkel, Markus von Flüe, Luigi M Terracciano, Salvatore Piscuoglio, Charlotte K Y Ng

**Affiliations:** Department of Bioinformatics, Institute of Biochemistry and Biophysics, University of Tehran, Tehran, Iran; Institute of Medical Genetics and Pathology, University Hospital Basel, Basel, Switzerland; Institute of Medical Genetics and Pathology, University Hospital Basel, Basel, Switzerland; Visceral Surgery and Precision Medicine Research laboratory, Department of Biomedicine, University of Basel, Basel, Switzerland; Institute of Medical Genetics and Pathology, University Hospital Basel, Basel, Switzerland; Visceral Surgery and Precision Medicine Research laboratory, Department of Biomedicine, University of Basel, Basel, Switzerland; Department of Bioinformatics, Institute of Biochemistry and Biophysics, University of Tehran, Tehran, Iran; Visceral Surgery and Precision Medicine Research laboratory, Department of Biomedicine, University of Basel, Basel, Switzerland; Institute of Medical Genetics and Pathology, University Hospital Basel, Basel, Switzerland; Department of Biosystems Science and Engineering, ETH Zurich, Basel, Switzerland; SIB Swiss Institute of Bioinformatics, Basel, Switzerland; Novartis Institutes for BioMedical Research, Novartis Pharma AG, Basel, Switzerland; Novartis Institutes for BioMedical Research, Novartis Pharma AG, Basel, Switzerland; Institute of Medical Genetics and Pathology, University Hospital Basel, Basel, Switzerland; Clarunis, Department of Visceral Surgery, University Centre for Gastrointestinal and Liver Diseases, St. Clara Hospital and University Hospital Basel, Switzerland; Department of Biosystems Science and Engineering, ETH Zurich, Basel, Switzerland; SIB Swiss Institute of Bioinformatics, Basel, Switzerland; Clarunis, Department of Visceral Surgery, University Centre for Gastrointestinal and Liver Diseases, St. Clara Hospital and University Hospital Basel, Switzerland; Department of Pathology, Humanitas Clinical and Research Center, IRCCS, Milan, Italy; Department of Biomedical Sciences, Humanitas University, Milan, Italy; Institute of Medical Genetics and Pathology, University Hospital Basel, Basel, Switzerland; Visceral Surgery and Precision Medicine Research laboratory, Department of Biomedicine, University of Basel, Basel, Switzerland; Institute of Medical Genetics and Pathology, University Hospital Basel, Basel, Switzerland; Department for BioMedical Research, University of Bern, Bern, Switzerland; SIB Swiss Institute of Bioinformatics, Lausanne, Switzerland

## Abstract

Systematic perturbation screens provide comprehensive resources for the elucidation of cancer driver genes. The perturbation of many genes in relatively few cell lines in such functional screens necessitates the development of specialized computational tools with sufficient statistical power. Here we developed APSiC (*A*nalysis of *P*erturbation *S*creens for *i*dentifying novel *C*ancer genes) to identify genetic drivers and effectors in perturbation screens even with few samples. Applying APSiC to the shRNA screen Project DRIVE, APSiC identified well-known and novel putative mutational and amplified cancer genes across all cancer types and in specific cancer types. Additionally, APSiC discovered tumor-promoting and tumor-suppressive effectors, respectively, for individual cancer types, including genes involved in cell cycle control, Wnt/β-catenin and hippo signalling pathways. We functionally demonstrated that *LRRC4B*, a putative novel tumor-suppressive effector, suppresses proliferation by delaying cell cycle and modulates apoptosis in breast cancer. We demonstrate APSiC is a robust statistical framework for discovery of novel cancer genes through analysis of large-scale perturbation screens. The analysis of DRIVE using APSiC is provided as a web portal and represents a valuable resource for the discovery of novel cancer genes.

## INTRODUCTION

Advances in large-scale functional screening technologies have enabled the discovery of gene requirements across diverse cancer entities (1,2). Systematic perturbation screens using short hairpin RNA (shRNA) or CRISPR are increasingly used to investigate how genetic alterations or expression modulation of individual genes lead to phenotypic changes, revealing novel factors in carcinogenesis (3–5). In parallel, large-scale sequencing efforts of 10000+ cancers have provided a comprehensive molecular portrait of human cancers and their molecular pathogenesis (6). Among the major findings is the unbiased discovery of genes mutated at rates significantly higher than the expected background level (7), revealing the global landscape of genetic ‘driver genes’ (8–11). The discovery of these ‘driver genes’ forms the critical foundations of cancer diagnostics, therapeutics, clinical trial design and selection of rational combination therapies. Despite the large cohort size, the functional consequences of mutations in rarely mutated genes, such as *YAP/TAZ* (12), are only revealed by functional studies. Moreover, a systematic survey of effectors, defined as genes whose tumor-promoting or tumor-suppressive properties are non-genetically driven (e.g. transcriptionally or epigenetically dysregulated genes), is lacking.

In the recent large-scale perturbation screen of the project DRIVE (deep RNAi interrogation of viability effects in cancer), 7837 genes in 398 cancer cell lines were targeted with a median of 20 shRNAs per gene across a variety of malignancies to generate a comprehensive atlas of cancer dependencies (4). The findings described in the study provided an overarching view of the nature and types of cancer dependencies, but has barely scratched the surface of the potential of the data generated. This huge resource of deep, robust and well-curated functional data, in conjunction with molecular profiling from the Cancer Cell Line Encyclopedia (CCLE) (13), remains a largely untapped resource to be thoroughly mined and interrogated.

Analysis of shRNA perturbation screens is challenging due to the off-target effects of shRNAs as well as the low number of cell lines screened (4,14). Project DRIVE used deep coverage libraries to alleviate the off-target issues (4). Additionally, several computational tools have been developed to elicit the on-target effect from RNAi screens that have both on- and off-target effects (14–18). RSA provides an absolute gene score based on the rank distribution of phenotypes produced by all shRNA reagents of a given gene (17). ATARiS estimates a consensus shRNA profile for each gene and provides a relative gene score (16). DEMETER is a regularized linear model for computing the gene effects due to knockdown of a target gene by taking into account the off-target effects due to the seed sequence (14). DEMETER2 is a complex hierarchical model structure that provides absolute gene dependency scores as opposed to ATARiS and DEMETER that provide relative dependency scores (18).

Cancer dependencies can be divided into two main groups of non-self dependencies (or synthetic lethality) and self-dependencies. Synthetic lethality refers to the cell dependency on the concomitant loss of two or more genes. In the context of an shRNA or CRISPR screen, synthetic lethality refers to the loss of cell viability upon knockdown/knockout of a gene conditional on the loss-of-function state of another gene. Various computational approaches have been used to discover synthetic lethal pairs from perturbation studies (19–21). MiSL is a computational pipeline for the identification of synthetic lethal pairs using Boolean implications applied to the The Cancer Genome Atlas (TCGA) datasets (22,23). A recent computational tool, called SLIdR, uses Irwin-Hall tests and causal inference for the discovery of pan-cancer as well as cancer-specific synthetic lethal pairs using the DRIVE data (24). On the other hand, self-dependency refers to cell dependency on a gene with specific molecular features such as mutation, copy number, expression, and DNA methylation. In the original DRIVE project, univariate *k*-means clustering with *k* = 3 was used for each gene to divide cell lines into three sets (4). Various statistical tests such as Fisher's Exact and Wilcoxon tests, and differential expression analysis were then used to discover association between molecular features and gene dependencies for the two extreme sets of cell lines. In a recent study, the Pearson's correlation test was used to find the association between gene dependency and molecular features (25). These approaches for finding self-dependencies are mainly applicable to the pan-cancer setting where a sufficient number of cell lines is available (4), as they lack statistical power for a limited number of observations (i.e. 5–10 observations). While project DRIVE is one of the largest of its kind, the number of cell lines for some cancer types is still quite small. A tool that can identify self-dependencies with small sample size is needed to make the best use of perturbation screens to reveal the cancer type-specific vulnerabilities.

Here, we introduce APSiC (*A*nalysis of *P*erturbation *S*creens for *i*dentifying novel *C*ancer genes), a novel tool for the systematic and robust interrogation of large-scale perturbation screens to discover self-dependencies even with limited number of samples. Incorporating mutation and copy number status of the samples, APSiC identifies potential genetic drivers and effectors. We considered three classes of genetic drivers, namely missense mutational cancer genes, non-missense mutational cancer genes and amplified cancer genes, as well as two classes of effectors namely tumor-promoting and tumor-suppressive effectors. Of particular importance, effectors identified by APSiC have been largely under-studied in cancer research. We applied APSiC to the DRIVE dataset and identified both known and novel candidate genetic drivers and effectors in 26 cancer types. As a proof of concept, we functionally validated *LRRC4B* as a putative tumor-suppressive effector in breast cancer. We provided the statistical analysis of DRIVE by APSiC as a web portal (https://apsic.scicore.unibas.ch/) for the scientific community to explore and functionally characterize genes that may be involved in carcinogenic processes and may pave the way for the discovery of novel cancer-related biomarkers and drug targets.

## MATERIALS AND METHODS

### The APSiC algorithm

**Figure 1.**
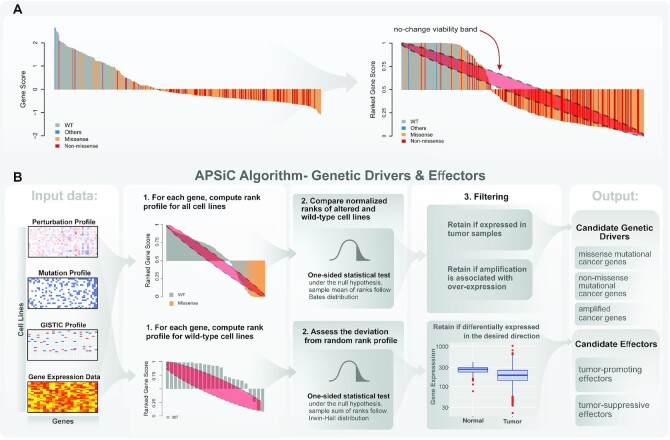
Overview of the APSiC algorithm. (**A**) Illustration of the transformation from the raw cell viability scores for a given gene (left, using *TP53* as an example) to the rank profile (right). Each bar of the waterfall plots represents one sample and is colored by the mutational status of the given gene in the sample. The red ellipse in the rank profile (right) represents a no-change (random) viability band. (**B**) Schematic representation of the APSiC algorithm for identifying genetic drivers and effectors (See Materials and Methods for details).

In this section, we give a technical description of APSiC and introduce a new waterfall plot, called rank profile, for the visualization of gene dependencies in knockdown screens. We considered the knockdown experiments of *p* genes across *N* cell lines. Let ${\nu _{ij}}$ be viability of cell line $i \in \{ {1, \cdot \cdot \cdot ,\ N} \}$ upon knocking down gene $j \in \{ {1,\ \ldots ,\ p} \}$ and ${m_{ij}}$ be a binary variable indicating whether a specific genetic alteration (i.e. mutation or copy number alteration) is present in gene *j* of cell line *i*. In this study, we only considered missense and non-missense (i.e. nonsense, insertions and deletions, splice site and mutations affecting start or stop codons) mutations. Waterfall plots are often used to show viabilities of knockdown experiments for a single gene across different cell lines. As an example, the waterfall plot for the gene *TP53* is shown in Figure 1A (left). Each vertical bar corresponds to a cell line and is colored by the mutation type present in *TP53*. Figure 1A indicates that cell lines with the presence of missense or non-missense mutations in *TP53* tend to have lower viabilities upon knockdown of this gene. While the waterfall plot is a useful visualization tool for demonstrating gene dependencies, it lacks sufficient interpretability in certain cases, particularly when the number of cell lines is limited. In this paper, we introduce a new waterfall plot, named rank viability profile or simply rank profile, to address this issue.

To make viability scores comparable across cell lines, we computed normalized rank values per cell lines denoted as ${r_{ij}}$, representing the rank of viability for gene *j* among all knockdown experiments in cell line *i*. For mathematical convenience and without loss of generality, we normalized ranks to the range of [0, 1]. When the number of knockdown genes is high, normalized ranks have many distinct levels in the interval [0, 1] and we assumed normalized ranks are continuous. Let ${R_{1,A}},\ {R_{2,A}},\ \ldots ,\ {R_{N,A}}$ denote random variables associated to the ranks of gene *A* in *N* cell lines. We dropped subscript A and denoted ranks as ${R_1},\ {R_2},\ \ldots ,\ {R_N}$ for the simplicity of notation. By placing ranks ${R_i}$ in ascending order and renaming them, we obtained ${Y_1} < {Y_{2\ }} < \ldots < {Y_N}$ where ${Y_i}$ is called *i*th ordered statistic. It is easy to see that ${Y_1} = \mathop {min}\limits_{} \ ( {{R_1},\ \ldots ,\ {R_N}} )$ and ${Y_N} = \mathop {max}\limits_{} \ ( {{R_1},\ \ldots ,\ {R_N}} )$. The probability density function of ordered statistic ${Y_i}$ in general is given as

$$\begin{equation*}f\ \left( {{y_i}} \right) = \ Nf\left( r \right)\left( {\begin{array}{@{}*{1}{c}@{}} {N - 1}\\ {i - 1} \end{array}} \right)F{\left( r \right)^{i - 1}}{\left( {1 - F\left( r \right)} \right)^{N - i}}\end{equation*}$$

where *f*(*r*) and *F*(*r*) denote the probability density and the cumulative distribution functions, respectively. If there is no dependency between knocking down of a gene and the viability of the cell, we can assume ${R_i} \sim U( {0,\ 1} )$ for $i\ = \ 1,\ \ldots ,\ N$, hence we have ${Y_i} \sim Beta( {i,\ N - i + 1} )$. Using this result, we constructed a no-change viability band at statistical significance *α* using the quantiles of ${Y_i}$ at the $\alpha /2$ and $1 - \alpha /2$ for $i\ = \ 1,\ \ldots ,\ N$. Now we defined a new plot, called rank viability profile or simply rank profile, as a waterfall plot using normalized ranks, realizations of ${R_i}$ for a gene, overlaid with the no-change viability band (Figure 1A).

The APSiC algorithm identifies potential cancer genes by assessing deviation of the respective rank profiles from what is expected by chance. The algorithm can identify genetic drivers and effectors (Figure 1B). We considered three categories for genetic drivers, namely

- Missense mutational cancer gene: defined as genes for which reduced viabilities are observed preferentially in samples with at least a missense mutation in the respective gene.
- Non-missense mutational cancer gene: defined as genes for which reduced viabilities are observed preferentially in samples harboring at least a non-missense mutation in the respective gene.
- Amplified cancer gene: defined as genes for which reduced viabilities are observed preferentially in samples with copy number amplification.

We considered two categories for effectors, namely

- Tumor-promoting effectors: defined as genes for which reduced viabilities are observed in samples without a genetic alteration in the respective gene.
- Tumor-suppressive effectors: defined as genes for which increased viabilities are observed in samples without a genetic alteration in the respective gene.

For genetic drivers, the APSiC algorithm considers rank profiles of mutated and wild-type (i.e. without non-synonymous mutation, copy number amplification or deep deletion) samples with respect to an input gene $g$ (Figure 1B). Then, it performs a one-sided statistical test to determine whether rank scores of the two groups of samples are significantly different in the direction of interest, according to the genetic feature of interest. Suppose $R_1^{wt},\ R_2^{wt},\ \ldots ,\ R_m^{wt}$ and $R_1^{mu},\ R_2^{mu},\ \ldots ,\ R_n^{mu}$ are random variables denoting rank scores upon knockdown of gene $g$ for $m$ wild-type and $n$ mutated samples, respectively. Let $\overline {{R^{wt}}}$ and $\overline {{R^{mu}}}$ denote the average ranks of the wild-type and mutated samples, respectively. We defined $S\ = \overline {{R^{mu}}} \ - \overline {{R^{wt}}}$ as the test statistic and ${s_{obs}}$ as the observed test statistic. We assumed the null hypothesis is that the knockdown of gene $g$ does not have an impact on the cell viability; in other words, there is no difference in the average ranks of the two groups, i.e. $S\ =\ 0$. The general formula for the distribution of any weighted sum of uniform random variables is given in (26). We simplified the general formula and obtained the null distribution of the test statistic $S$ as

$$\begin{eqnarray*}P({S \le \ s})&=&\frac{(-1)^m}{n^n m^m(n+m)!}\sum_{k=0}^{n}\sum_{p=0}^{m}(-p)^{p+k}\binom{n}{k}\binom{m}{p}\\ &&\times\left(s+\frac{k}{n}-\frac{p}{m}\right)^{m+n}\theta\left(s+\frac{k}{n}-\frac{p}{m}\right) \end{eqnarray*}$$

where

$$\begin{equation*}\theta (t) =\left\{\begin{matrix} 0 & t < 0 \\ \frac{1}{2} & t = 0 \\ 1 & t >0 \end{matrix}\right. \end{equation*}$$

For mutational and amplified cancer genes, we computed lower-tailed *P*-values, $P( {S \le {s_{obs}}} )$. Due to numerical issues, it is impractical to use the exact null distribution formula for large values of *m* and *n* (*m + n* > 20). In this case, we computed an approximation for the null distribution of *S* as follows. Under the null hypothesis, $\overline {{R^{wt}}}$ and $\overline {{R^{mu}}}$ follow the *Bates* distributions *Bates(m)* and *Bates(n)*, respectively. The Bates distribution is a distribution that represents the mean of a number of independent uniform random variables on the unit interval. For large values of *m* and *n*, $\overline {{R^{wt}}}$ and $\overline {{R^{mu}}}$ are approximately distributed by $N( {\frac{1}{2}\ ,\ \frac{1}{{12m}}} )$ and $N( {\frac{1}{2}\ ,\ \frac{1}{{12n}}} )$. Hence under the null hypothesis, the test statistic *S* is approximately distributed as $N( {0,\ \frac{1}{{12}}( {\frac{1}{m} + \frac{1}{n}} )} )$. Additionally, given that mutational cancer genes are not expected to be functional unless the gene is expressed in the given cancer type, we required the putative mutational cancer genes to be expressed in cancer samples. Similarly, amplified cancer genes are not expected to be functional unless the amplification is associated with overexpression. Hence, we required the expression levels of a putative amplified cancer gene to be higher in cancer samples where the given gene is amplified in comparison with non-amplified samples using the one-sided Wilcoxon rank sum test. *P*-value ≤0.05 was considered to be statistically significant.

To identify effectors, we considered the wild-type (i.e. without non-synonymous mutation, copy number amplification or deep deletion) samples with respect to an input gene $g$ (Figure 1B). The null hypothesis is that the knockdown of gene *g* does not have an impact on cell viability. We defined the test statistic as $T\ = \ {R_1} + {R_2} + \ldots + {R_m}$ and ${t_{obs}}$ as the observed test statistic. Under the null hypothesis, *T* follows an Irwin-Hall distribution $T \sim IH( m )$, which represents the summation of $m$ independent uniform random variables on the unit interval. For large values of *m*, *T* is approximately distributed as $N( {m/2,\ m/12} ).$ To identify tumor-promoting effectors, we required significant lower-tailed *P*-values, $P( {T\ \le {t_{obs}}} )$ for wild-type cell lines with respect to the input gene. Additionally, for the respective tissue type, the overall expression at the RNA level of a tumor-promoting effector in tumor samples was required to be significantly higher than that in normal tissue samples using the Wilcoxon rank sum test. On the contrary, for identifying tumor-suppressive effectors, we required significant upper-tailed *P*-values, $P( {T \ge {t_{obs}}} )$, for wild-type cell lines with respect to the input gene as well as lower RNA expression of tumor samples in comparison to normal tissue samples using the Wilcoxon rank sum test. *P*-value ≤ 0.05 was considered to be statistically significant.

### Downloading and preprocessing of DRIVE, CRISPR and TCGA data

**Figure 2.**
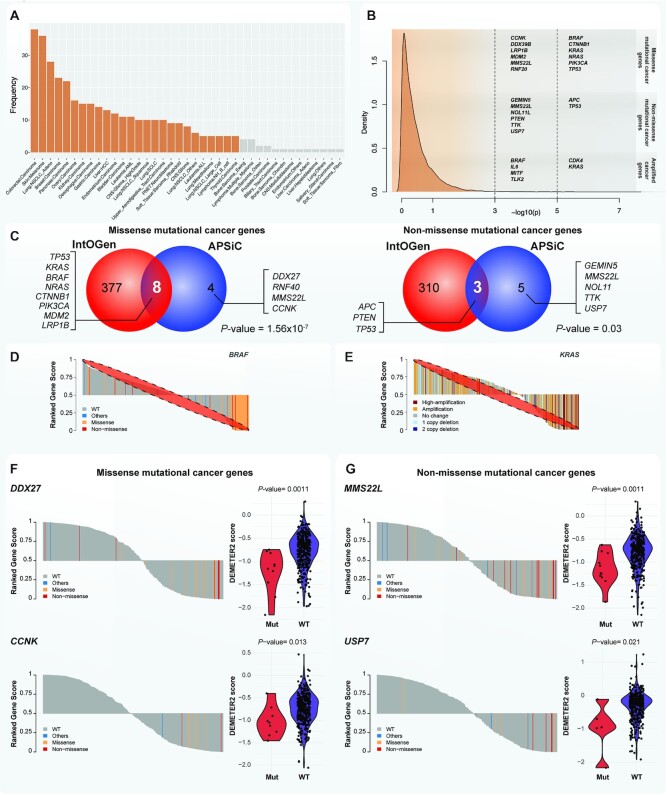
Pan-cancer analysis of genetic drivers in the DRIVE perturbation screen. (**A**) The number of cell lines available for the 39 cancer types in the DRIVE perturbation screen. Orange bars indicate cell lines for which > 4 cell lines are available for cancer type-specific analyses. (**B**) Kernel density estimation of the *P*-values (on a -log_10_ scale) for genetic drivers using the APSiC algorithm in a pan-cancer analysis. Candidate mutational cancer genes and amplified cancer genes identified by the APSiC, reaching significance level after accounting for multiple testing, are shown. (**C**) Venn diagram showing the overlap of the pan-cancer (left) missense mutational cancer genes/(right) non-missense mutational cancer genes and IntOGen mutational driver genes. It is noteworthy the latest release of the IntOGen (v20200201) comprises 568 cancer driver genes, a subset of which were not used for the Venn diagrams due to the lack of experiment in the DRIVE project or preprocessing filters. In particular, 183 and 255 IntOGen genes were excluded from this analysis for missense and non-missense mutational cancer genes, respectively. The number of genes that are not in APSiC mutational cancer genes and IntOGen mutational driver genes is 5316 and the number of genes that are not in APSiC non-missense mutational cancer genes and IntOGen mutational driver genes is 3073. *P*-value was computed by the hypergeometric test. (**D**) Rank profile for a missense mutational cancer gene (*BRAF*) colored by mutation status. (**E**) Rank profile of an amplified cancer gene (*KRAS*) colored by copy number status. (**F, G**) Rank profiles of selected novel pan-cancer (**F**) missense mutational cancer genes (*DDX27* and *CCNK*) and (**G**) non-missense mutational cancer genes (*MMS22L* and *USP7*). To the right of the rank profiles are the DEMETER2 absolute gene scores of the corresponding genes.

We considered the viability profiles of 372 cell lines in the project DRIVE (4) for which their genetic profiles (somatic mutations and GISTIC2 copy number alterations) were available at the Cancer Cell Line Encyclopedia (Figure 2A) (13). We used aggregated gene-level viability scores for each experiment by the RSA and ATARiS algorithms (16,17). The RSA and ATARiS scores were available for 7837 and 6729 genes, respectively. We used the same method as defined in the project DRIVE to remove essential genes, defined as genes with an RSA value of ≤3 in more than half of the cell lines (4). After the removal of 174 essential genes and removing genes without GISTIC2 (copy number alterations) profiles, ATARiS scores were available for 6203 genes in 372 cell lines in 39 cancer types. We also obtained the DEMETER2 absolute dependency scores (18), available for 5898 of the 6203 genes. The CRISPR data was available at the DepMap project (3). In total, we considered CERES viability scores of 536 cell lines for which genetic profiles were available; out of which 270 cell lines were included in the DRIVE project.

TCGA gene expression, mutation and GISTIC2 data were obtained for the 21 cancer types for which data are available for cancer samples and for the 13 cancer types for which gene expression data are available for cancer and normal tissues (Supplementary Figure S1). The data were downloaded using the TCGAbiolinks and the cBioPortal packages (27,28). zFPKM transformation was used to define genes undergoing active gene expression, where a threshold of –3 was previously shown to differentiate between active and background gene expression (29). Genes were defined as expressed if zFPKM > –3 in >50% of the samples. The normalized expression level of genes in FPKM (Fragments Per Kilobase of transcript per Million mapped reads) were used for the identification of effectors.

### Analysis of DRIVE and CRISPR validation sets by APSiC

The primary APSiC analysis was performed on the ATARiS scores from DRIVE. For the identification of the missense mutational cancer genes, samples with *Missense_Mutation* were considered in the mutant class. For the identification of non-missense mutational cancer genes, samples with the following non-missense mutations were considered in the mutant class: *In_Frame_Ins*, *In_Frame_Del*, *Frame_Shift_Ins*, *Frame_Shift_Del*, *Nonsense_Mutation*, *Splice_Site*, *Start_Codon_Del*, *Stop_Codon_Del*, *Stop_Codon_Ins*, *Start_Codon_Ins*. Similarly for amplified cancer genes, samples with GISTIC2 (30) copy number state 2 (amplified) were considered in the mutant class. For the mutation-level analysis, samples harboring a specific *Missense_Mutation* were considered in the mutant class. For the APSiC analyses focusing only on homozygous mutations, we defined a missense mutation as homozygous if it is accompanied with a non-missense mutation or loss of heterozygosity. Similarly, a non-missense homozygous mutation is defined as a non-missense mutation in conjunction with a second non-missense mutation or loss of heterozygosity. In all analyses of the genetic drivers and effectors, samples that did not harbor non-synonymous mutations and did not harbor copy number amplification (GISTIC2 copy number state 2) or deep deletion (GISTIC2 copy number state –2) with respect to the input gene were considered in the wild-type class.

For the pan-cancer analyses, we included all 372 cell lines in 39 cancer types. In the analyses of individual cancer types, we considered the 26 cancer types for which more than four cell lines are available in the DRIVE data. For the identification of pan-cancer and cancer-specific genetic drivers, we considered only genes for which there were at least two samples harboring a genetic alteration of the corresponding class and at least two wild-type samples. For the effectors, we considered genes for which there were at least two wild-type samples for the gene.

To cross-validate our results with the DepMap CRISPR data, for the genes identified as significant in the APSiC analysis of the DRIVE data, we performed APSiC analyses on the CERES viability scores, separately for all cell lines with CERES data, for the subset of cell lines with CERES data and are in DRIVE, and for the subset of cell lines with CERES data but are not in DRIVE.

### Multiple testing

To address the multiple comparisons problem, we chose a significance level such that the expected number of false positives due to multiple testing for each cancer and feature is equal to one. To this end, we chose a significance level of $1/n$, or 0.05 if $1/n$>0.05, where $n$ is the number of genes tested for identification of drivers. This approach is less conservative than other correction methods such as the Bonferroni and Benjamini-Hochberg methods and allows us to retain interesting hits while keeping the number of false positives low.

### Statistical analysis

The catalogue of mutational driver genes was obtained from IntOGen (9) (v20200201, the compendium of cancer genes). Enrichment analysis was performed by the hypergeometric test. Wilcoxon rank sum test was used to compare DEMETER2 viability scores between wild-type and mutated cell lines.

To identify potential upstream genetic alterations for tumor-promoting and tumor-suppressive effectors, we performed analyses by comparing the genetic profiles of cancers in the TCGA cohort with higher versus lower expression levels of a putative effector using the chi-squared test. *P*-values were corrected for multiple testing using the Benjamini-Hochberg approach (Supplementary Methods).

### Transient gene knockdown by siRNAs and transient gene overexpression

Breast cancer-derived cell lines (MCF-7, BT-549 and MDA-MB231, Supplementary Methods) were used for transient gene knockdown by siRNA (BT-549 and MDA-MB231) or transient gene overexpression (MCF-7).

Transient gene knockdown was conducted using ON-TARGET plus siRNA transfection. ON-TARGET plus SMARTpool siRNAs against human *LRRC4B* (Dharmacon, CO; #L-023786-01-0005), ON-TARGET plus SMARTpool non-targeting control and DharmaFECT transfection reagent (Dharmacon, CO; #T-2001-03) were all purchased from GE Dharmacon. ON-TARGET plus SMARTpools comprise 4 individuals siRNAs and were chosen over single siRNAs to minimize off-target effects (31–33). Transfection was performed according to the manufacturer's protocol (Supplementary Methods).

For gene overexpression, log-phase MCF-7 breast cancer cells were seeded in 6-well plates at approximately 50–60% confluence and transfected with pLV-EGFP:T2A:Puro-CMV > Luc2 (Vectorbuilder, #VB190320-1059xxv) or pLV-EGFP:T2A:Puro-CMV > hLRRC4B (NM_00108457.1) (Vectorbuilder, #VB190321-1196fvk) expression vectors. Transfection medium was replaced after 8 h.

### Protein extraction and western blot

Protein extraction and western blots were performed as previously described (34) with modifications (Supplementary Methods). The ratio of proteins of interest/loading control in treated samples were normalized to their counterparts in control cells. Antibodies against LRRC4B (PA5-23529, Thermofisher) and B-actin (A5441, Sigma) were used at dilution 1:1000 and 1:5000, respectively.

### Proliferation assay

Cell proliferation was assayed using the xCELLigence system (RTCA, ACEA Biosciences, San Diego, CA, USA) as previously described (35) (Supplementary Methods). The impedance signals were recorded every 15 min until 96/120 h and expressed as cell index values, calculated automatically and normalized by the RTCA Software Package v1.2. The values were defined as mean ± standard deviation. Multiple *t*-test analysis was performed using the GraphPad software.

### Migration assay

Migration assays were performed using the CIM-plate of the xCELLigence Real-Time Cell Analysis (RTCA, ACEA Biosciences, San Diego, CA, USA) system (Supplementary Methods). Measurements were taken every 15 min until 24 h after seeding and expressed as cell index values. Multiple *t*-test analysis was performed using the GraphPad software.

### Cell cycle and apoptosis analyses by flow cytometry

For cell cycle analysis, 72 h after transfection, cells were collected, stained with DAPI and analyzed by flow cytometry. For apoptosis analysis, BT-549 and MDA-MB231 cells were transfected with siRNA (control or against *LRRC4B*) and MCF-7 cells were transfected with *LRRC4B* overexpressing plasmid or control plasmid. 8 h after transfection, medium was changed and doxorubicin was added according to the respective IC50 for each cell line (36,37). 48 h post treatment with doxorubicin, cells were collected and stained with annexin V (Annexin V-FITC conjugate; Invitrogen, CO; #V13242) and propidium iodide (PI; Invitrogen, CO; #V13242), and analyzed by flow cytometry using the BD FACS Canto II cytometer (BD Biosciences, USA, Supplementary Methods). Data was analyzed using the FlowJo software version 10.5.3 (https://www.flowjo.com).

## RESULTS

### The APSiC algorithm

APSiC uses rank-based statistics to discover self-dependencies in perturbation screens (see Materials and Methods for a technical description of the algorithm). Given the raw cell viability readout of a perturbation screen, APSiC computes a rank profile for each gene by first ranking all genes by their viabilities upon knockdown in a given sample to the range of [0, 1] then aggregating the normalized ranks for a given gene across all samples (Figure 1A). Thus ranks close to zero represent reduced viability while the ranks close to one indicate cell growth upon knockdown. Incorporating mutation and copy number status of the samples, APSiC identifies potential genetic drivers and effectors by assessing the deviation of the distribution of normalized ranks from what is expected by chance using the Bates and Irwin-Hall tests. The Irwin-Hall distribution has been successfully used in identifying synthetic lethal gene pairs (24) and prioritizing cancer genes based on multi-omics data (38). The use of the rank-based statistics with the Bates and Irwin-Hall distributions provides enhanced statistical power when the number of samples is limited.

We considered three classes of genetic drivers (missense mutational cancer genes, non-missense mutational cancer genes and amplified cancer genes) and two classes of effectors (tumor-promoting effectors and tumor-suppressive effectors). We defined missense mutational, non-missense mutational and amplified cancer genes as genes for which reduced cell viabilities are preferentially observed in samples with missense mutations, non-missense mutations and copy number amplifications, respectively. To identify such genetic drivers, we tested, for a given gene, whether the ranks of the samples with and without the specific class of genetic alteration were significantly different using a one-sided Bates test, computing the lower-tailed *P*-values (i.e. the ranks preferentially suggest reduced viability upon gene knockdown, Figure 1B). We further evaluated the expression of the genes in human cancers and retained mutational cancer genes that are expressed in human cancers and amplified cancer genes for which amplification is associated with overexpression. For the effectors, we tested whether gene knockdown in samples without genetic alteration in the gene had any impact on cell viability by computing lower and upper-tailed Irwin-Hall test *P*-values for tumor-promoting and tumor-suppressive effectors, respectively (Figure 1B). We further tested whether the gene expression of candidate effectors was respectively enhanced or repressed in human cancers compared to the corresponding normal tissue type.

### APSiC identifies well-known and novel genetic cancer drivers

We applied APSiC to the DRIVE perturbation screens and the genetic data from the CCLE (13) to identify genetic driver genes. The dataset consists of 372 cell lines across 39 cancer types, with a median of 8 (range 1–38) cell lines per cancer type (Figure 2A). In a pan-cancer analysis, APSiC identified 12 missense mutational cancer genes, including the well-known mutational driver genes *TP53*, *KRAS*, *BRAF, NRAS, CTNNB1* and *PIK3CA* (Figures 2B–D, Supplementary Tables S1 and S2). Additionally, *DDX27, MDM2, RNF40, MMS22L, CCNK and LRP1B* were detected as missense mutational cancer gene (Figure 2F, Supplementary Figure S2 and Supplementary Tables S1 and S2). *MDM2* and *LRP1B* have recently been reported as mutational cancer drivers (9,39). Overall, we observed an enrichment of previously reported mutational driver genes among our missense mutational cancer genes (*P*-value = 1.56e–07, hypergeometric test, Figure 2C). On the other hand, *DDX27, RNF40, MMS22L* and *CCNK* have not been reported as mutational cancer genes in previous studies (9,10,39–42). Compared to the dN/dS ratio (i.e. the ratio between non-synonymous vs synonymous mutations, where non-synonymous refers to missense mutations here) of the known mutational cancer drivers (median 9.7, range 3.9–42.4), the four novel genes had lower dN/dS ratio (median 3.3, range 1.6–5.5, Supplementary Table S1). We noted that the dN/dS ratio for three of the four novel missense mutational cancer genes were within the range of or close to the range of the known mutational cancer drivers. Across the TCGA cohort, missense mutations in these four genes were found in 0.4–1.4% of the patients. We further assessed the DEMETER2 absolute gene dependency scores between the cell lines with missense mutations and the wild-type cell lines. For three of the four novel genes, knockdown of the genes in the wild-type cell lines reduced the cell viability modestly, while knockdown in the mutant cell lines further reduced the cell viability (Figure 2F and Supplementary Figure S2).

We identified eight non-missense mutational cancer genes, three of which were the classical tumor suppressor genes (TSGs) *TP53*, *APC* and *PTEN* (Figures 2B-C and Supplementary Tables S1-2). The remaining five were *TTK*, *MMS22L*, *GEMIN5*, *USP7* and *NOL11* (Figure 2G and Supplementary Figure S2). We observed that the non-missense mutational cancer genes were also enriched among previously reported mutational cancer drivers (*P*-value = 0.03, hypergeometric test, Figure 2C). For *TP53*, *APC* and *PTEN*, non-missense mutations were observed in 6.3–16.3% of the TCGA samples. For the remaining five genes, non-missense mutations were observed in <1% of the TCGA samples. In terms of dN/dS ratio, where non-synonymous refers to non-missense mutations, the five novel genes had lower dN/dS ratio (median 2.3, range 0.9–2.7 versus median 13.3, range 2.7–21.6 in the known cancer drivers, Supplementary Table S1). Similar to the novel missense mutational cancer genes, we observed that gene knockdown in the mutant cell lines enhanced the reduction in cell viability compared to gene knockdown in the wild-type cell lines (Figure 2G and Supplementary Figure S2).

The top amplified cancer genes were *KRAS, CDK4, BRAF, IL6, TLK2* and *MITF* (Figures 2B, E and Supplementary Tables S1 and S2). *MITF* is annotated in the Cancer Gene Census (11) as an amplification driver gene and a likely oncogenic driver in OncoKB (43). In OncoKB but not the Cancer Gene Census, amplifications of *CDK4* and *BRAF* are annotated as oncogenic whereas amplification of *KRAS* is likely oncogenic. By contrast, *TLK2 and IL6* have not been reported as an amplification driver in either the Cancer Gene Census or OncoKB and they are amplified in 1–2% of the TCGA cohort. The putative drug target *TLK2* is a nuclear serine/threonine kinase whose amplification has been shown to induce genomic instability by impairing the G2-M checkpoint and to enhance invasiveness (44,45). Overexpression of *IL6* leads to the activation of Jak/Stat signaling, which promotes tumorigenesis and tumor progression (46,47). Specifically, gene amplification of *IL6* has been associated with poor patient survival in glioblastoma (48).

*In silico* inference of the functional impact of mutations is typically based on observed frequency (10,40), positional clustering on the sequence level (49) or in the protein structure (50). Given that one of the main strengths of APSiC is the identification of dependencies in small sample sets, we hypothesize that APSiC could also be applied to discover cancer-associated mutations, specifically activating missense mutations clustered at specific amino acids (51). We therefore evaluated the DRIVE data on individual missense mutations. In the pan-cancer analysis, we identified 31 cancer-associated missense mutations in five genes (Supplementary Figure S3 and Supplementary Table S3). These mutations affect the previously reported hotspots (9,52) of *BRAF* (p.V600), *KRAS* (p.G12, p.G13, p.Q61), *NRAS* (p.G12, p.Q61), *PIK3CA* (p.E545, p.H1047), *TP53* (22 positions, including p.R175, p.R248 and p.R273). Notably we identified *KRAS* missense mutations at p.V14, immediately adjacent to the canonical p.G12/p.G13 hotspots. Compared to p.G12/p.G13, mutations at *KRAS* p.V14 are rare (0.01% in TCGA, compared to 6.3% p.G12 and 1% p.G13). In a series of 13336 colorectal cancers, p.V14 was identified as a *KRAS* mutation hotspot and p.V14 mutations have previously been associated with a range of RASopathies (53,54), supporting our finding that *KRAS* p.V14 mutations are rare but oncogenic.

### APSiC identifies cancer type-specific genetic cancer drivers

**Figure 3.**
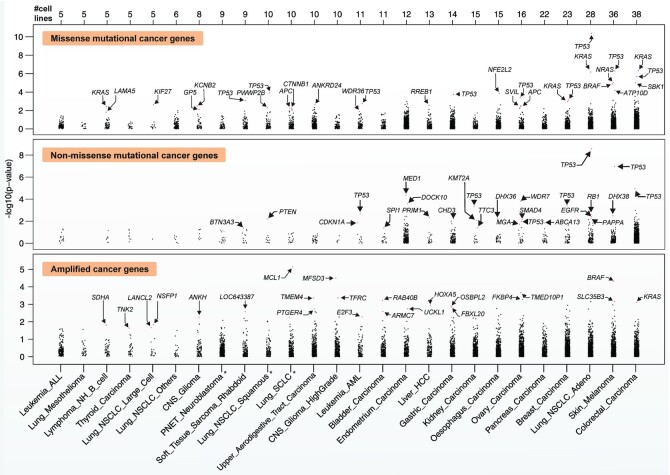
Cancer type-specific analysis of genetic drivers in the DRIVE perturbation screen. Dot plots of the *P*-values (on a -log_10_ scale) for genetic drivers using the APSiC algorithm in a cancer type-specific analysis, for (top) missense mutational cancer genes, (middle) non-missense mutational cancer genes and (bottom) amplified cancer genes. Genes reaching significance level after accounting for multiple testing are labelled. Cancer types are sorted by the number of cell lines. * denotes cancer types with no corresponding TCGA copy number and gene expression data.

We applied APSiC to the DRIVE data to identify genetic driver genes for individual cancer types. Across the 26 cancer types with more than four cell lines, we found 19 unique missense mutational cancer genes in 17 cancer types, 20 non-missense mutational cancer genes in 16 cancer types and 24 amplified cancer genes in 16 cancer types (Figure 3 and Supplementary Table S4). While the number of missense and non-missense mutational cancer genes correlated with the number of cell lines (*r* = 0.44, *P*-value = 0.03 and *r* = 0.79, *P*-value = 1.31e–6, respectively, both Spearman correlation tests), the number of amplified cancer genes did not (*r* = –0.004, *P*-value = 0.99, Spearman correlation test).

Three genes were identified as missense mutational cancer genes in multiple cancer types: *TP53* (nine cancer types)*, KRAS* (four), and *APC* (two). The other 16 genes were identified as missense mutational cancer genes in a single cancer type each. These included previously described mutational oncogenes *NFE2L2* in esophagus carcinoma, *BRAF* and *NRAS* in melanoma and *CTNNB1* in non-small cell lung cancer, all of which have been reported in IntOGen (9) as mutational drivers in their respective cancer types. The remaining 12 putative missense mutational cancer genes have not been reported as frequently mutated in human cancers. Given that missense mutations are not expected to be functional if the gene is not expressed in the corresponding cancer type, we further evaluated gene expression for the 21 cancer types for which corresponding TCGA gene expression data for cancers are available (Supplementary Figure S1). By computing zFPKM to define active gene expression (Materials and Methods and Supplementary Figure S4), we observed that *HNF4G*, identified in acute myeloid leukemia, is not expressed and therefore likely to be a false positive. For the remaining 11 genes, nine (*ATP10D*, *GP5*, *KCNB2*, *KIF27*, *PWWP2B, RREB1*, *SBK1, SVIL* and *WDR36)* were found to be expressed in their corresponding cancer types and two (*ANKRD24* and *LAMA5)* did not have gene expression data. These putative novel missense mutational cancer genes include *RREB1* (Ras Responsive Element Binding Protein 1, in hepatocellular carcinoma), which is a gene previously found to be significantly mutated in pancreatic carcinoma (10) and dysregulated in cancer (55). *SBK1* (SH3 Domain Binding Kinase 1), a novel missense mutational cancer gene in colorectal carcinoma, has also been previously found to be dysregulated in cancer (56). Three of the novel missense mutational cancer genes were mutated at >1% in their respective TCGA cancer types: *ATP10D* (9.4% in melanoma), *SVIL* (3.2% in ovarian carcinoma) and *WDR36* (3.2% in bladder carcinoma, Supplementary Table S4).

For the non-missense mutational cancer gene, *TP53* was identified in seven cancer types (Figure 3 and Supplementary Table S4). Our candidates also included several genes typically considered TSGs, including *CDKN1A* (bladder carcinoma, 9.2% in TCGA), *KMT2A* (kidney carcinoma, 0.6%), *MGA* (ovarian carcinoma, 1.5%), *PTEN* (high-grade glioma, 14.2%), *RB1* (lung adenocarcinoma, 5.0%), *SMAD4* (ovarian carcinoma, 0.5%). We also found *EGFR* (lung adenocarcinoma, 5.1%), where the two cell lines harbored canonical activating in-frame deletions in exon 19 (57). Of those not previously reported in IntOGen as mutational driver genes, all were found to be expressed in their corresponding cancer types and *MED1* and *DOCK10* were found to be mutated at >1 in endometrial cancers.

The 24 amplified cancer genes were identified in 14 cancer types (Figure 3 and Supplementary Table S4). All amplified cancer genes were identified in single cancer types. Analogous to the additional filtering of the mutational cancer genes by gene expression, amplified genes are not expected to be functional unless they are overexpressed. We therefore evaluated gene expression and copy number data for the 21 cancer types for which corresponding TCGA data for cancers are available (Supplementary Figure S1). Focusing on the 13 genes for which the association between gene amplification and overexpression could be assessed, 12 were found to show overexpression when amplified. Aside from *KRAS* (colorectal cancer, amplified in 1% of TCGA cases), *MCL1* (lung squamous carcinoma, 4.7%) and *BRAF* (melanoma, 4.1%) amplifications have also been implicated as oncogenic alterations in OncoKB (43). Five genes are amplified in > 5% of the corresponding TCGA cohorts: *E2F3* (cytoband 6p22.3, 15.7% of bladder cancer), *TFRC* (3q29, 13.0% of head and neck cancers), *MFSD3* (8q24.3, 7.9% of head and neck cancers, *FKBP4* (12p13.33, 6.8% of ovarian carcinoma) and *FBXL20* (17q12, 6.4% of gastric cancer).

In our analysis of cancer-associated missense mutations, aside from the mutations in *BRAF*, *KRAS*, *NRAS*, *PIK3CA* and *TP53*, we identified the *FGFR2* p.N549 mutation in endometrial carcinoma (2.0% in TCGA) and the *CTNNB1* p.G34 mutation in gastric carcinoma (0.7% in TCGA), both of which are known activating hotspot mutations (9,58) (Supplementary Table S3). The remaining putatively cancer-associated missense mutations have not been observed in the corresponding TCGA cohorts.

Taken together, APSiC identified well known and putative genetic driver genes not only in pan-cancer analysis but also in individual cancer types, even cancer types with small sample size.

### Survey of effectors reveals cancer type specificity

**Figure 4.**
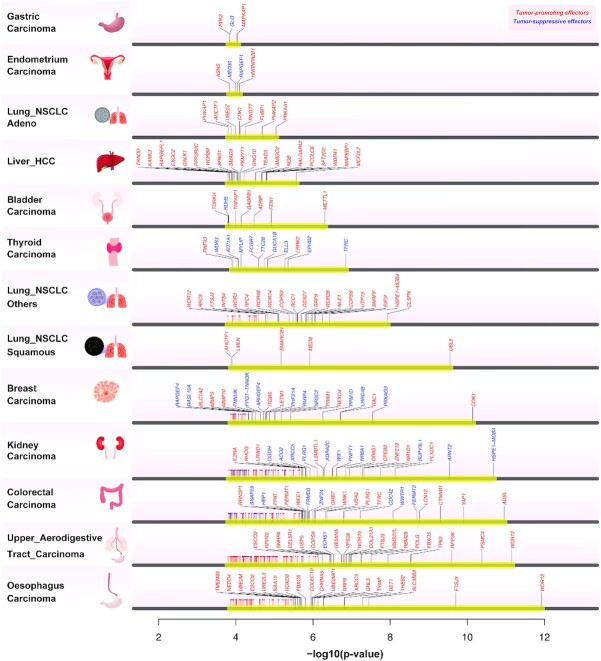
Cancer type-specific analysis of the effectors in the DRIVE perturbation screen. Effectors identified in the 13 cancer types with corresponding gene expression data from the TCGA. APSiC *P*-values are shown (on a –log_10_ scale) for effectors significant after multiple testing corrections and over- or under-expressed in human cancers for tumor-promoting effectors (in red) and tumor-suppressive effectors (in blue), respectively. The top 20 genes for each cancer type are labelled.

Previous studies have reported lineage-specific dependencies where some genes are specifically essential for specific cancer types (1). Using the APSiC effector models, which recognize irregular viability patterns in the rank profiles of cell lines wild-type for a given gene (Figure 1B), we indeed observed that cancer types in the DRIVE dataset showed diverse pattern of dependencies, with a strong separation between epithelial and non-epithelial cancers (Supplementary Figure S5, Supplementary Table S5 and Supplementary Methods). We therefore analyzed the DRIVE data with the APSiC effector models to identify such lineage-specific effectors for individual cancer types. Based on the DRIVE screen alone, we identified a median of 27 tumor-promoting effectors (range 3–542) and 34 tumor-suppressive effectors (range 0–459) per cancer type. However, we reasoned that genuine tumor-promoting and tumor-suppressive effectors would also be over- and under-expressed, respectively, in the corresponding human cancer types. Therefore, for the 13 cancer types for which gene expression data for the cancer and corresponding non-cancer counterparts were available from the TCGA (Supplementary Figure S1), we further restricted the putative tumor-promoting and tumor-suppressive effectors to those that were over- and under-expressed, respectively, relative to their non-cancer counterparts. After this filtering step, there were a median of 21 tumor-promoting effectors (range 2–106) and two tumor-suppressive effectors (range 0–45, Figure 4) per cancer type.

We identified several well-known oncogenes as tumor-promoting effectors, including *CDK1* (breast cancer and lung adenocarcinoma), *WEE1* (colorectal cancer), *RAC1* (breast cancer), *CTNNB1* (colorectal cancer), *LGR5* (breast cancer), *YAP1* (colorectal cancer) and *WWTR1* (kidney cancer), all of which are frequently overexpressed in diverse types of cancers. *CDK1* and *WEE1* are regulators of the cell cycle. In particular, *CDK1* plays a critical role in the control of cell division (59,60) and *WEE1* controls the G2/M checkpoint in response to DNA damage (61). Overexpression of *RAC1*, a member of the Rac family small GTPases, is associated with oncogenic transformation and cell invasion (62). *CTNNB1* and *LGR5* are components of the canonical Wnt/β-catenin signalling pathway that, when activated, leads to the transcription of oncogenic target genes such as c-MYC and cyclin D1 (63). *YAP1* and *WWTR1* (also known as TAZ) are core members of the Hippo signaling pathway that plays a key role in biological processes such as cell proliferation, survival and differentiation and whose deregulation leads to oncogenesis (64). Related to Hippo signaling, we also identified *TEAD3*, a lesser described member of the TEAD family involved in hippo signalling, as a tumor-promoting effector in liver cancer (65). Among the tumor-suppressive effectors, there were a number of known or putative TSGs. For instance, *RAPGEF1* (also known as C3G, endometrial cancer) is frequently hypermethylated in cervical carcinoma and consequently inactivated (66).

Our results here show that APSiC identified effectors in individual cancer types. However, we also note many genes, in particular the tumor-suppressive effectors, have not been associated with carcinogenesis.

### *LRRC4B* is a putative tumor suppressor gene in breast cancer

**Figure 5.**
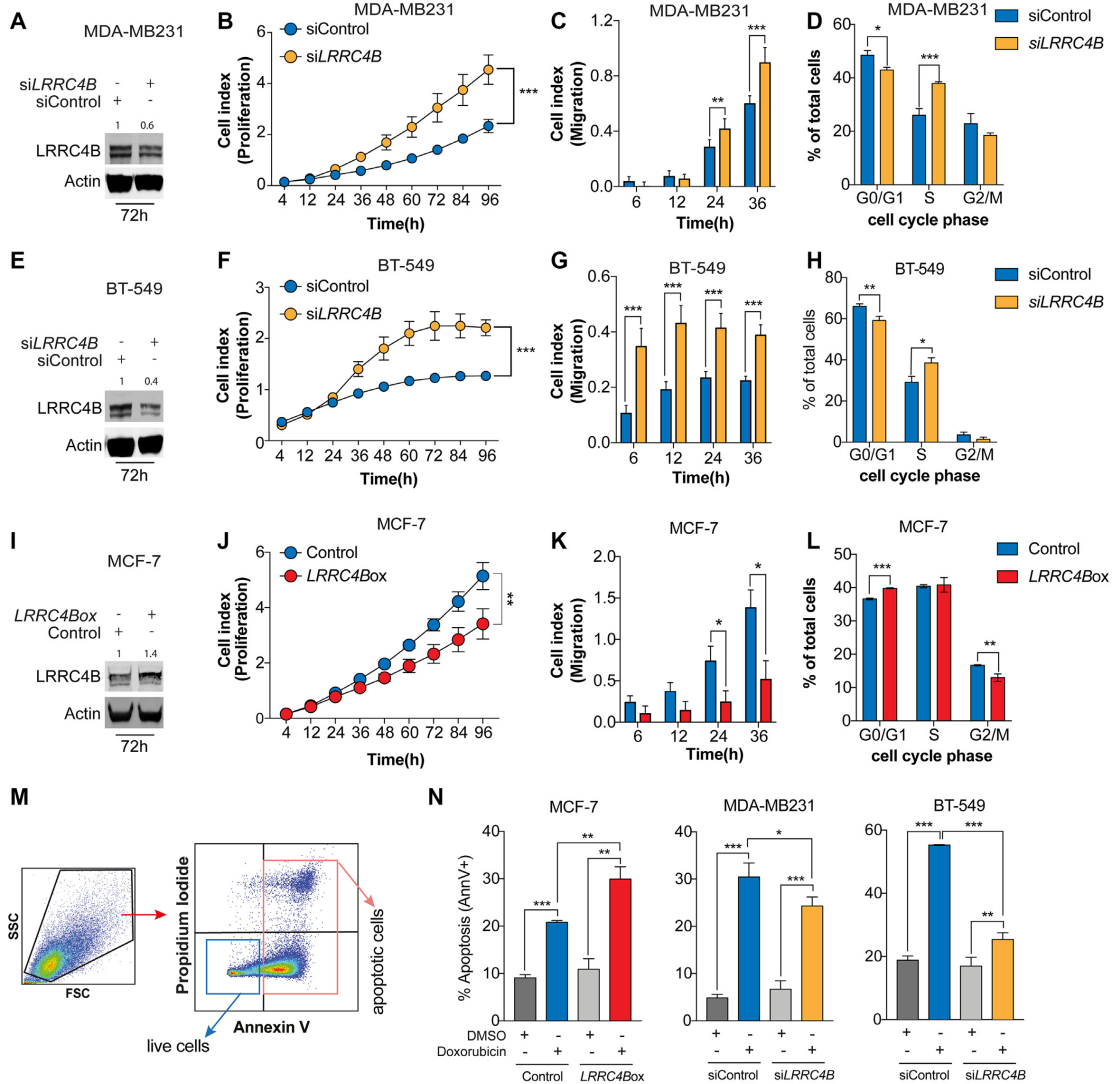
*LRRC4B* has tumor suppressor-like properties in *in vitro* models of breast cancer. (**A, E, I**) Western blotting showing LRRC4B protein level in (**A**) MDA-MB231, (**E**) BT-549 and (**I**) MCF-7 cell lines 72 h post-transfection. Quantification is relative to the loading control (actin) and normalized to control sample. (**B, F, J**) Proliferation kinetics of (**B**) MDA-MB231, (**F**) BT-549 and (**J**) MCF-7 cells upon (**B, F**) downregulation or (**J**) upregulation of *LRRC4B* compared with the control. (**C, G, K**) Migration potential of (**C**) MDA-MB231, (**G**) BT-549 and (**K**) MCF-7 cells upon (**C, G**) downregulation or (**K**) upregulation of *LRRC4B* compared with the control. (**D, H, L**) Cell cycle analysis of (**D**) MDA-MB231, (**H**) BT-549 and (**L**) MCF-7 cells upon (**D, H**) downregulation or (**L**) upregulation of *LRRC4B* compared with the control. (**M**) Dot plot illustrating the flow cytometry gating strategy used to assess cell viability and apoptosis using Annexin V and propidium iodide staining of MDA-MB231, BT-549 and MCF-7 cells upon downregulation/ upregulation of *LRRC4B* compared with the control cells, with and without Doxorubicin. (**N**) Quantification of the mean (±SD) percentage of apoptotic cells (AnnV+) across the different groups. Error bars represent standard deviation obtained from three independent experiments. For all experiments, statistical significance was assessed by multiple *t*-tests (* *P* < 0.05, ** *P* < 0.01, *** *P* < 0.001).

As a proof-of-concept to validate APSiC, we selected *LRRC4B*, a top putative tumor-suppressive effector in breast cancer, for functional validation. A literature search of *LRRC4B* in cancer suggests that its role in carcinogenesis is unknown. In the TCGA cohort, overexpression of *LRRC4B* was associated with an enrichment of *CDH1* somatic mutations and a depletion of *TP53* mutations (Supplementary Table S4). Meanwhile, one of its paralogs *LRRC4* has been shown to have a putative tumor suppressor role in glioma by modulating the extracellular signal-regulated kinase/protein kinase B/nuclear factor-κB pathway (67). In the DRIVE RNAi screen, nearly all breast cancer cell lines displayed significantly increased cell viability upon *LRRC4B* knockdown (Supplementary Figure S6A) and breast cancers in TCGA showed lower *LRRC4B* expression compared to normal breast tissue (Supplementary Figure S6B). We selected the breast cancer cell lines MDA-MB231, BT-549 and MCF-7 with high, moderate and low endogenous *LRRC4B* expression to investigate whether modulating *LRRC4B* expression would result in other classical cancer phenotypes such as increased migration and colony formation (Supplementary Figure S6C). We silenced *LRRC4B* in MDA-MB231 and BT-549 using siRNA, reducing LRRC4B protein expression by 40% and 60%, respectively, 72 h post-transfection (Figures 5A, E and Supplementary Figure S6D). In both models, *LRRC4B* downregulation significantly increased the proliferation and migration rates (Figures 5B, C and F, G). By contrast, *LRRC4B* overexpression significantly reduced proliferation and migration in MCF-7 (Figures 5I-K and Supplementary Figure S6D).

*LRRC4* has been shown to suppress cell proliferation by delaying cell cycle in the late G_1_ phase (68,69). To test whether *LRRC4B* may play the same role in breast, we analyzed cells with *LRRC4B* overexpression or downregulation stained with DAPI by flow cytometry (FACS, Supplementary Figure S6E). *LRRC4B* knockdown in MDA-MB231 and BT-549 promoted cell transition into the S phase (Figures 5D, H), while *LRRC4B* overexpression in MCF-7 significantly retained cells in the G_1_ phase (Figure 5L), suggesting a similar mechanism to *LRRC4*.

A common mechanism of oncogenicity is resistance to apoptosis (70). To test whether modulation of apoptosis is a mechanism of action of *LRRC4B* as an oncosuppressor, we induced apoptosis with doxorubicin and measured it using Annexin V and propidium iodide co-staining followed by FACS analysis (Figure 5M). Forty eight hours after treatment, *LRRC4B*-overexpressing MCF-7 cells showed 10% more apoptotic and 10% fewer live cells, suggesting that *LRRC4B* overexpression could sensitize cells to doxorubicin-induced apoptosis (Figure 5N and Supplementary Figure S6F). By contrast, *LRRC4B-*downregulating MDA-MB231 and BT-549 cells showed increased resistance to doxorubicin and had 25% and 10% fewer apoptotic and 25% and 10% more live cells, respectively (Figure 5N and Supplementary Figure S6F). Our results provide compelling evidence that APSiC identified *LRRC4B* as a novel oncosuppressor gene in breast cancer.

## DISCUSSION

While large-scale perturbation screens such as DRIVE include cell lines from various cancer types, the identification of cancer dependencies was performed at the pan-cancer level due to the limited number of samples for individual cancer types (4). This limitation is due to the fact that computation models such as *k*-means clustering in conjunction with classical statistical tests (e.g. Fisher's exact test) are not statistically powerful enough to discover potential cancer drivers and effectors in the low sample size setting (4). In this study, we addressed this limitation by introducing APSiC, a novel statistical testing tool for the systematic and robust interrogation of large-scale perturbation screens applicable at the level of individual cancer types. APSiC is based on the Bates and Irwin-Hall distributions that can identify potential genetic drivers and effectors even with a limited number of samples.

Our pan-cancer analysis for genetic drivers identified a number of classical mutational driver genes such as *KRAS*, *NRAS*, *BRAF*, *CTNNB1*, *PIK3CA*, *TP53, APC* and *PTEN*, as well as amplified driver genes such as *MITF, BRAF* and *CDK4*. Here we identified *TP53* as both a missense and non-missense mutational cancer gene. While *TP53* has traditionally been considered a TSG, studies have reported that many of its missense mutations are oncogenic (71–73) by, for example, altering tumor cell biology through their interaction with other transcription factors or co-factors (74,75). Similarly, while *APC* is a classical TSG in colorectal cancers, frequently harboring truncating variants (41), here we also found that *APC* is a missense mutational cancer gene in ovarian carcinoma and lung squamous cell carcinoma. While colon tumors are mainly predominated by frameshift and stop gains, 47.1% of *APC* mutations in gastric cancer are missense (76). A missense mutation in the *APC* gene has also been reported in pancreatoblastoma and shown to exert reduced repression on Wnt/β-catenin signaling (77). Our analysis of the amplified cancer genes also identified probable drivers *TLK2 and IL6*, whose amplification and/overexpression have been implicated in genomic instability (44,45) and Jak/Stat activation (46,47), respectively.

One could speculate that cell lines with mutations may be more dependent on the second wild-type allele for survival (i.e. haploinsufficiency, Supplementary Tables S1 and S4). Focusing on cell lines with heterozygous mutations, we found that six pan-cancer missense mutational cancer genes (*TP53*, *KRAS*, *BRAF*, *CTNNB1*, *PIK3CA* and *LRP1B*) remained significant hits when we only considered cell lines with homozygous deletions. On the other hand, with few cell lines with homozygous mutations, *NRAS* and *MMS22L* were not significant, and the remaining four were not evaluable (insufficient number of cell lines with homozygous mutations). Of the eight pan-cancer non-missense mutational cancer genes, three (*TP53*, *APC* and *PTEN*) remained significant while the remaining five were not evaluable. For the cancer type-specific analyses, all evaluable missense and non-missense mutational cancer genes remained significant considering only cell lines with homozygous mutations (*n* = 23, 34 not evaluable). Our results suggest that for the genes frequently associated with the loss of the wild-type allele, haploinsufficiency alone does not explain our observations.

Our analysis of the DRIVE data used the ATARiS scores, which are gene scores relative to all screened cell lines estimated from the consensus shRNA profile for each gene. Thus neither ATARiS nor the rank profile from APSiC provide information into whether absolute cell viability was actually increasing or decreasing upon knockdown. The DRIVE screen has since been summarized as DEMETER2 absolute gene scores, where a value >0 indicates an absolute cell growth upon gene inhibition and *vice versa* (18). Evaluating the DEMETER2 absolute gene scores in our pan-cancer APSiC analysis of genetic drivers, we found that for 11/12 missense mutational cancer genes, 8/8 non-missense mutational cancer genes and 6/6 amplified cancer genes, the mutant cell lines showed median negative DEMETER2 scores, indicating an absolute reduction of cell growth upon gene inhibition (Supplementary Table S1). On the other hand, wild-type cell lines with respect to 335/385 (87%) of the tumor-promoting effectors and 71/131 (54%) of the tumor-suppressive effectors displayed decreased and increased, respectively, cell viability. Of note, *LRRC4B*, a tumor-suppressive effector in breast cancer, did not show absolute increased cell numbers upon inhibition according to DEMETER2. However, we note that our *in vitro* experiments demonstrated that *LRRC4B* was, in fact, a tumor-suppressive effector.

On the basis of the DEMETER2 absolute gene scores, for 3/4 novel missense mutational cancer genes (*DDX27*, *CCNK*, *RNF40*) and 5/5 novel non-missense mutational cancer genes (*TTK*, *GEMIN5*, *MMS22L*, *USP7*, *NOL11*), gene knockdown in the wild-type cell lines reduced cell viability while in the mutant cell lines, gene knockdown further reduced cell viability. There could be many possible interpretations of the observations. For instance, one could speculate that these non-missense mutations could result in a change or gain of function, leading to a dependency on the presence of these mutations. On the other hand, one could also explain the observations by hypothesising that these mutations may be hypomorphic, which would lead to a partial loss of protein function. While gene knockdown by shRNA in the wild-type cell lines may not completely deplete gene expression and result in the partial loss of cell viability, gene knockdown in the context of a hypomorphic mutation may result in (near) complete silencing and therefore (nearly) completely abrogate gene function and cell viability. One could also speculate that in the context of a hypomorphic mutation, the (near) complete silencing may result in catastrophic events (e.g. acute genomic instability) that may result in cell death. We also cannot rule out the possibility that these genes may include essential genes. However, we note that essential genes may also act as functional drivers, as demonstrated for *ADSL* (78), a tumor-promoting effector in colorectal cancer (Supplementary Table S4).

While the DRIVE RNAi screen is the largest, and arguably most robust, loss-of-function screen to date, it has since been complemented by the CRISPR–Cas9 loss-of-function screen of the Dependency Map (DepMap) project (3). To evaluate whether our APSiC (DRIVE) hits could be validated in the DepMap CRISPR screen, we analyzed the CRISPR data with APSiC for the significant hits identified in the DRIVE RNAi screen, separately for cell lines included (n = 270) and not included (*n* = 266) in the DRIVE screen and altogether (*n* = 536) (Supplementary Figure S7 and Supplementary Table S6). On the pan-cancer level, 7/12 missense mutational cancer genes were also significant hits in at least one of the three analyses of the DepMap CRISPR screen while only *TP53* was identified as a significant non-missense mutational cancer gene in the DepMap CRISPR screen. For the individual cancer types, 8/26 missense mutational cancer genes and 4/19 non-missense mutational cancer genes were confirmed in the DepMap CRISPR screen. In terms of the effectors, 601/1685 (36%) of the APSiC (DRIVE) tumor-promoting effectors and 327/1502 (22%) of the tumor-suppressive effectors were confirmed in the APSiC analysis of the CRISPR screen in at least one subset of cell lines. We did not assess cross-validation for amplified cancer genes as CRISPR screens were known to have gene-independent response in copy number amplified regions (79). Although CRISPR–Cas9 may show superior specificity compared to RNAi, the DRIVE RNAi screen is arguably more robust given that it used a median of 20 shRNAs per gene, while the CRISPR–Cas9 screen used only a median of 4 sgRNAs per gene, summarized using the CERES model (3). CERES is a computation scheme for the inference of gene-dependency scores from CRISPR–Cas9 screens by correcting for the gene-independent copy-number effects on CRISPR–Cas9 targeting. From a biological perspective, CRISPR–Cas9 screens are knockout screens that completely deplete gene expression as opposed to shRNA screens, which are knockdown screens that result in partial reduction of expression. This difference likely accounts for some of the discrepancies observed as well. Thus there are fundamental differences between the ATARiS and the CERES data, on the biological, experimental and statistical perspectives.

Our analysis of the DRIVE data showed a significant enrichment for known mutational driver genes. However, our analysis did not identify a number of known mutational cancer drivers, for example, *CTNNB1* in hepatocellular carcinoma, *PIK3CA* in breast cancer and *NFE2L2* in lung squamous cell carcinoma. We note that our dataset did not have any *CTNNB1*-mutant hepatocellular carcinoma cell lines. For *PIK3CA* in breast cancer, the APSiC missense mutational cancer gene *P*-value was 0.01, but did not reach significance level after accounting for multiple testing. There were 11 *PIK3CA*-mutant breast cancer cell lines in the DRIVE dataset, with 4 harboring the canonical p.H1047R mutation, 2 p.E545K, 1 p.E542K, 2 p.C420R and 2 rare mutations of unknown significance. Previous studies have demonstrated that the phenotypic effects of *PIK3CA* mutations are not all equal, with p.E542K, p.E545K and p.H1047R showing stronger oncogenic properties compared to the rarer mutations (e.g. p.C420R) (80,81). This observation is also in agreement with our mutation-level analysis in breast cancer, in which we identified p.H1047 mutations as oncogenic while p.C420 was not, albeit with fewer mutants. For *NFE2L2* in lung squamous cell carcinoma, while the APSiC rank for the LK2 cells with the gain-of-function p.E79K mutation (82) was < 0.001, the APSiC rank for EBC1 with the rarer p.D77V mutation was 0.83, suggesting that p.D77V may not critical to the survival of the EBC1 cells. We note that while APSiC is a statistically robust framework, the results are dependent on the input data. Specifically in the context of the DRIVE dataset of cell lines, one could not control for the divergent genetic backgrounds between cell lines.

In conclusion, APSiC is a novel tool to enable systematic and robust discovery of gene dependencies from a small number of samples. Our analysis of the DRIVE perturbation screens using APSiC is a valuable resource for the discovery of drug targets, cancer-related biomarkers and novel cancer genes, in particular of effectors for which a systematic analysis has been lacking. Our results would complement the associated multi-omics profiling (25,83) and drug screens (84) to understand the vulnerabilities of cancer.

## DATA AVAILABILITY

The RSA and ATARiS gene scores have already been published as a part of the project DRIVE (https://data.mendeley.com/datasets/y3ds55n88r/4). GISTIC2 profiles of the cell lines are available at the CBioPortal website (https://www.cbioportal.org/study?id=cellline_ccle_broad). The mutation profiles, DEMETER2 data (version: 20Q2) and CRISPR (version: 20Q3) were downloaded from the DepMap data portal (https://depmap.org/portal/download/). The CRISPR data were obtained from the project Achilles (Achilles_gene_effect.csv). We used the TCGAbiolinks package in R (27) to download the TCGA gene expression and mutation data and the cBioPortal package in R (28) to download the GISTIC2 data (August/September 2020). The LOH information was obtained from the CCLE ABSOLUTE copy number analysis results (CCLE_ABSOLUTE_combined_20181227.csv, DepMap project, CCLE 2019 release).

The code for the APSiC algorithm is available at https://github.com/hesmon/APSiC/. A web portal using the Shiny framework in R has been developed to visualize rank profiles of the DRIVE shRNA screen and corresponding gene expression data from TCGA at https://apsic.scicore.unibas.ch/, where the complete set of results supporting the conclusions of this article can also be downloaded.

## Supplementary Material

gkab627_Supplemental_Files
