# Supplementary material for: Systematic identification of novel cancer genes through analysis of deep shRNA perturbation screens
Source: Nucleic Acids Res. 2021 Jul 27;49(15):8488–504. doi: 10.1093/nar/gkab627 (PMC8421231; doi:10.1093/nar/gkab627)
Supplement: gkab627_Supplemental_Files [file gkab627_supplemental_files.zip › Montazeri et al. Supplementary Data 1.pdf]

SUPPLEMENTARY FIGURES

Supplementary Figure S1

Pathological annotation in the DRIVE project	TCGA project	Pathological annotation in the DRIVE project	TCGA project
Oesophagus Carcinoma*	ESCA^*	Bladder Carcinoma	BLCA^*
Upper Aerodigestive Tract Carcinoma*	HNSC^*	Liver_HCC	LIHC^*
Lung_NSCLC Squamous*	LUSC^*	Thyroid Carcinoma	THCA^*
Colorectal Carcinoma*	COAD^* READ^*	Kidney Carcinoma	KIRC^*
Gastric Carcinoma*	STAD^*	Lung_NSCLC Adeno Lung_NSCLC Others	LUAD^*
Breast Carcinoma*	BRCA^*	Endometrium Carcinoma*	UCEC^*
Lymphoma NH_B_cell	DLBC^	Leukemia:AML	LAML^
CNS:Glioma	LGG^	Lung Mesothelioma	MESO^
Ovary Carcinoma	OV^	Pancreas Carcinoma	PAAD^
Skin Melanoma	SKCM^	Soft Tissue Sarcoma Rhabdoid	SARC^
CNS Glioma_HighGrade	GBM^	Lung_NSCLC Large_Cell, Leukemia_ALL PNET Neuroblastoma, Lung_SCLC	Not Available

^ gene expression, mutation and GISTIC2 data available for cancers
* gene expression data available for cancer and normal tissues

Supplementary Figure S1: Pathological annotation in the DRIVE project and the corresponding TCGA projects used for gene expression analysis.

Supplementary Figure S2

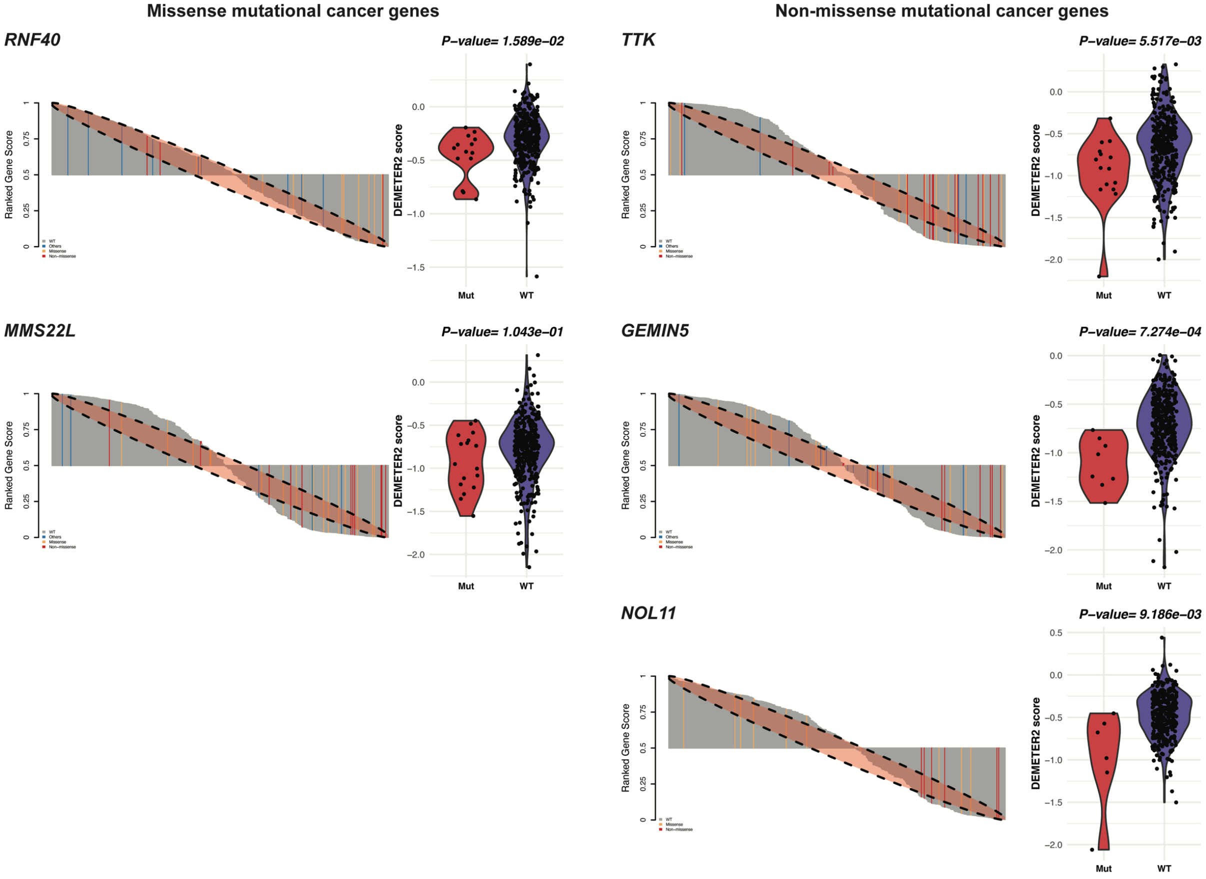

Supplementary Figure S2: Rank profiles (left) for the missense mutational cancer genes *RNF40* and *MMS22L* and the non-missense mutational cancer genes *TTK*, *GEMIN5* and *NOL11* in DRIVE and their associated DEMETER2 scores (right).

Supplementary Figure S3

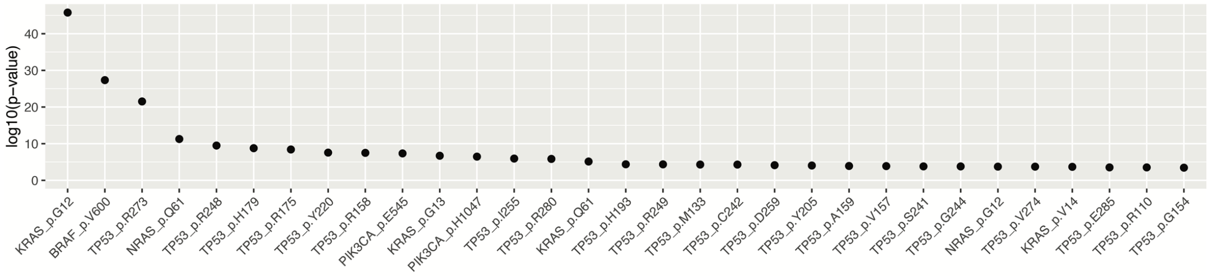

Supplementary Figure S3: Dot plot of the *P*-values (on a -$\log_{10}$ scale) for cancer-associated missense mutations using the APSiC algorithm in a pan-cancer analysis.

Supplementary Figure S4

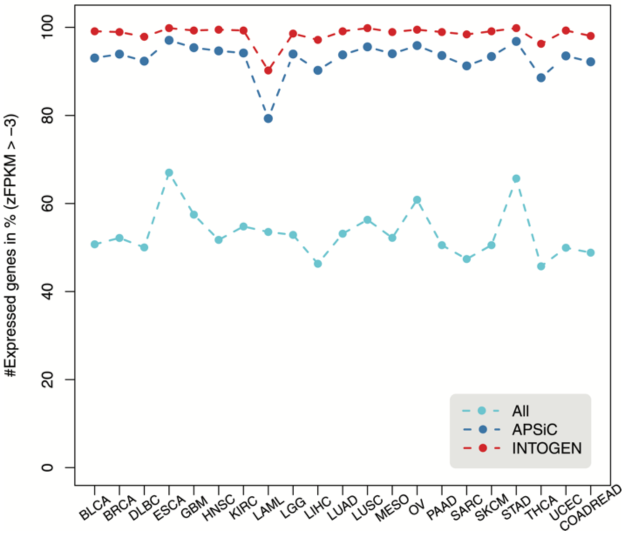

Supplementary Figure S4: Fraction of expressed genes, as defined as zFPKM > -3 in at least 50% of the samples in the TCGA gene expression datasets for all genes (cyan), genes included in the APSiC analysis of DRIVE (blue) and IntOGen genes (red). Note that genes included in the APSiC analysis were preferentially expressed compared to all genes in the TCGA datasets.

Supplementary Figure S5

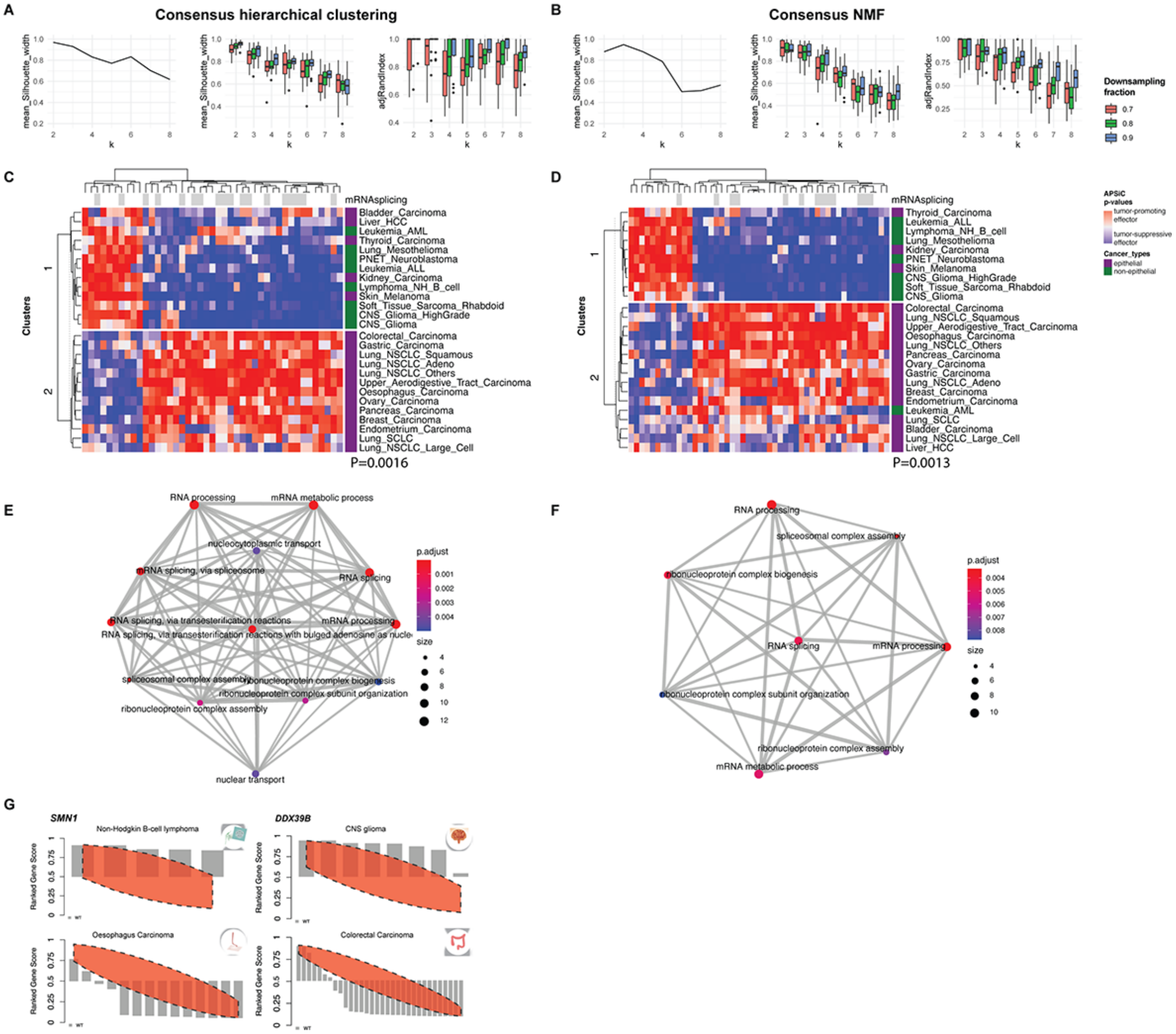

Supplementary Figure S5: Clustering of the 500 most variable *P*-values (in terms of median absolute deviation) for effectors using the APSiC algorithm for the 26 cancer types in the DRIVE perturbation screen. **A-B,** Mean Silhouette widths for (**A**) consensus hierarchical clustering and (**B**) consensus NMF clustering to identify the optimum number of clusters. Downsampling analysis to determine robustness of clusters. Both clustering identified 2 clusters across cancer types. **C-D,** Heatmaps illustrate the clusters identified by (**C**) consensus hierarchical clustering and (**D**) consensus NMF clustering. Heatmaps show the genes with differential dependency between the two clusters. Genes related to mRNA splicing are indicated in grey boxes above the heatmap. *P*-values refer to Fisher's exact tests performed to evaluate the enrichment of epithelial vs non-epithelial cancers between the clusters. **E-F,** Enrichment maps showing pathways enriched among genes that showed differential dependency between the two clusters in (**E**) consensus hierarchical clustering and (**F**) consensus NMF clustering. An enrichment of mRNA splicing-related genes was observed in both clustering. **G,** Rank profiles for *SMN1* in (top) non-Hodgkin B-cell lymphoma and (bottom) oesophagus carcinoma and for *DDX39B* in (top) CNS glioma and (bottom) colorectal carcinoma. *SMN1* (Survival Of Motor Neuron 1, Telomeric) and *DDX39B* (DExD-Box Helicase 39B) are both regulators of the spliceosome. See also **Supplementary Table S5** and **Supplementary Methods**.

Supplementary Figure S6

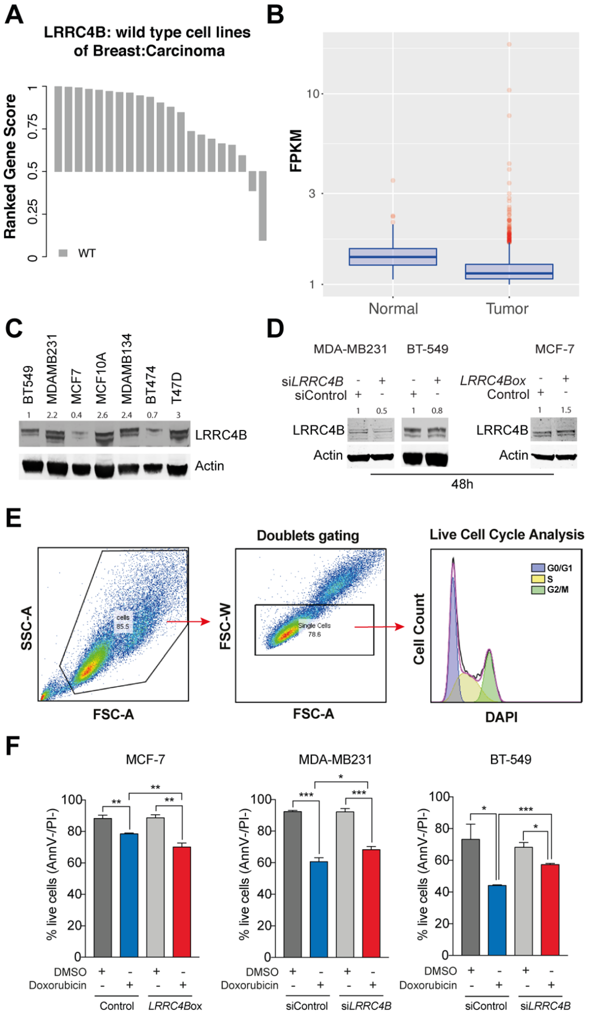

Supplementary Figure S6: **A,** Rank profile of the *LRRC4B* gene in breast cancer cell lines. Each bar in the waterfall plots represents one cell line and is colored by the mutation status. **B,** *LRRC4B* transcript expression in breast cancers and normal tissues. The plot was generated using gene expression data obtained from the TCGA dataset. **C,** Screening of LRRC4B protein expression in a panel of breast cancer cell lines by western blot. Quantification is relative to the loading control (actin) and shown as ratio above the western blot plot. **D,** Western blotting showing LRRC4B protein level in MDA-MB231, BT-549 and MCF-7 cell lines 48 hours post transfection. Quantification is relative to the loading control (actin) and normalized to control sample. **E,** Dot plot showing the flow cytometry gating strategy used to assess cell cycle status on breast cancer cell lines in both overexpressing and downregulating *LRRC4B* cells. (*** *P*-value < 0.001). **F,** Quantification of the mean (+/- SD) percentage of live cells (AnnV-/PI-) across the different groups. Error bars represent standard deviation obtained from three independent experiments. For all experiments, statistical significance was assessed by multiple t-tests (* *P*-value < 0.05, ** *P*-value < 0.01, *** *P*-value < 0.001).

Supplementary Figure S7

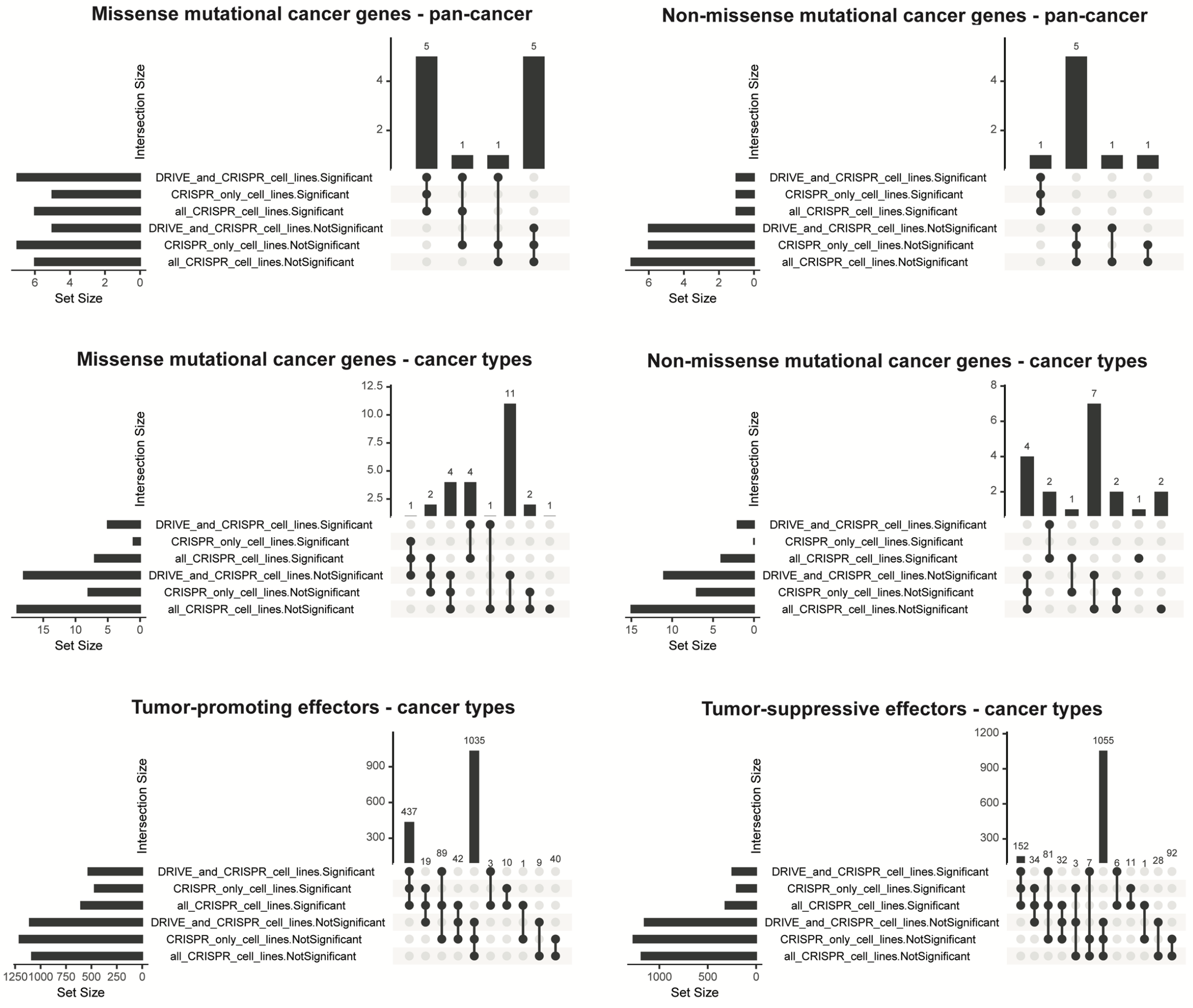

Supplementary Figure S7: UpSet plots show the number of significant APSiC genes in the CRISPR validation datasets. Horizontal bars represent the number of cell lines in the CRISPR validation dataset that are also in DRIVE (‘DRIVE_AND_CRISPR_cell_lines’), the number of cell lines in the CRISPR dataset only (i.e. not in DRIVE, ‘CRISPR_only_cell_lines’), and the number of cell lines in the complete CRISPR dataset (‘all_CRISPR_cell_lines’). The horizontal bars are further subdivided based on whether the APSiC analysis of the CERES scores on the selected (sub)sets of cell lines are significant. Vertical bars represent the number of genes in the intersection of the subsets (indicated by the black dots joined by black lines) shown on the horizontal bars.
