# Supplementary material for: Systematic identification of novel cancer genes through analysis of deep shRNA perturbation screens
Source: Nucleic Acids Res. 2021 Jul 27;49(15):8488–504. doi: 10.1093/nar/gkab627 (PMC8421231; doi:10.1093/nar/gkab627)
Supplement: gkab627_Supplemental_Files [file gkab627_supplemental_files.zip › Montazeri et al. Supplementary Materials.pdf]

Supplementary material for

Systematic Identification of Novel Cancer Genes through Analysis of Deep shRNA Perturbation Screens

Montazeri *et al.*

SUPPLEMENTARY METHODS

Clustering and pathway analysis

Clustering was performed using consensus clustering (1) and consensus non-negative matrix factorization (NMF) (2) using the CancerSubtypes Bioconductor package (3) for the 500 most variable features in terms of median absolute deviation. Missing values were imputed using the nearest neighbor averaging with the impute R library. Consensus clustering was performed using 1-Spearman correlation as the distance metric and the Ward hierarchical clustering algorithm with 100 subsamples. Consensus NMF was performed with 50 runs. For both methods, we evaluated up to 8 clusters and the number of clusters was determined based on the mean Silhouette widths. To ensure robustness of the clustering, we further performed 20 iterations of downsampling to 70%, 80% and 90% and evaluated the mean Silhouette widths and concordance with the clusters obtained from the full dataset using the adjusted Rand index. Differential gene features between clusters were identified by Wilcoxon test, corrected for multiple testing by the Benjamini-Hochberg method. Pathway analysis was performed by over-representation analysis using ClusterProfiler (4) for the biological processes of Gene Ontology.

Analysis of potential upstream genetic alterations for effectors

To identify potential upstream genetic alterations for tumor-promoting and tumor-suppressive effectors, we performed analyses by comparing TCGA genetic profiles of tumors with higher versus lower expression levels of a putative effector. We dichotomized the expression levels into low or high using the median of expression levels of the putative effector in tumor samples. We then assessed the association of mutation status of all genes to the expression levels of the putative effector using the chi-squared test. The p-values were corrected for multiple testing using the Benjamini-Hochberg approach. A gene is called enriched or depleted for the effector if it is mutated more in high or low expressed samples, respectively. All the analyses were separately performed for misense and deleterious mutations.

Cell lines

Breast cancer derived cell lines (MCF-7, BT-549 and MDA-MB231) were maintained in a 5% CO_2-humidified atmosphere at 37°C and cultured in DMEM supplemented with 10% FBS, 1% Pen/Strep (Bio-Concept) and 1% MEM-NEAA (MEM non-essential amino acids, ThermoFisher Scientific). All cell lines were confirmed negative for mycoplasma infection using the PCR-based Universal Mycoplasma Detection kit (American Type Culture Collection, Manassas, VA) as previously described (5).

Transient gene knockdown by siRNAs

Log-phase breast cancer cells were seeded at approximately 60% confluence. Because antibiotics affect the knockdown efficiency of ON-TARGET plus siRNAs, growth medium was removed as much as possible and replaced by antibiotic-free complete medium. siRNAs were added to a final concentration of 25 nM. Cells were incubated at 37°C in 5% CO_2 for 24-48-72

hours for 48-72 hours for protein analysis. To avoid cytotoxicity, the transfection medium was replaced with a complete medium after 8 hours.

Protein extraction and western blot

Proteins were extracted using Co-IP buffer (100 mmol/L NaCl, 50 mmol/L Tris pH 7.5, 1 mmol/L EDTA, 0.1% Triton X-100) supplemented with 1x protease inhibitors (cOmplete Mini, EDTA-free Protease Inhibitor Cocktail, Roche, CO, #4693159001) and 1x phosphatase inhibitors (PhosSTOP #4906837001, Merck). Cell lysates were then treated with 10x reducing agent (NuPAGE Sample Reducing Agent, Invitrogen, #NP0009), 4x loading buffer (NuPAGE LDS Sample Buffer, Invitrogen, #NP0007), boiled and loaded into neutral pH, pre-cast, discontinuous SDS-PAGE mini-gel system (NuPAGE 10% Bis-Tris Protein Gels, ThermoFisher). The proteins were then transferred to nitrocellulose membranes using the Trans-Blot Turbo Transfer System (Bio-Rad). The membranes were blocked for 1 hr with Sure Block (Lubio Science) and then probed with primary antibodies overnight at 4°C. Next day, the membranes were incubated for 1 hr at RT with fluorescent secondary goat anti-mouse (IRDye 680) or anti-rabbit (IRDye 800) antibodies (both from LI-COR Biosciences). Blots were scanned using the Odyssey Infrared Imaging System (LI-COR Biosciences) and band intensity was quantified using ImageJ software.

Proliferation assay

To evaluate cell proliferation using the xCELLigence system (RTCA, ACEA Biosciences, San Diego, CA, USA), cells were first seeded and transfected in 6 well plates and 24 h after transfection $5x10^3$ cells were resuspended in 100 µl of medium and plated in each well of an E-plate 16. Background impedance of the xCELLigence system was measured for 12 s using 50 µl of room temperature cell culture media in each well of E-plate 16. The final volume in each well was then 150 µl.

Migration assay

To evaluate cell migration using the xCELLigence system (RTCA, ACEA Biosciences, San Diego, CA, USA), cells were first transfected in 6-well plates and 24 h after transfection, they were harvested and seeded in the CIM-plate. Every well of the bottom chamber was filled with 160 µl of the corresponding medium at 10% FBS concentration. After placing the upper chamber on top of the lower chamber, 50 µl of serum free medium was added on each CIM well for the background measurement. After 3x PBS washing, $3x10^4$ cells re-suspended in 100 µl of the corresponding medium at 1% FBS concentration were seeded in each well of the upper chamber.

Cell cycle analysis

Seventy-two hour after transfection, cells were collected, stained with DAPI and analyzed by flow cytometry using the BD FACS Canto II cytometer (BD Biosciences, USA). Briefly cells were harvested and washed 2X in PBS to get rid of serum proteins at 1200 rpm for 5 minutes. Pellets (up to $3x10^6$ cells) were resuspended in 1.2 ml PBS (Ca and Mg free). For crosslinking proteins 3.0 ml of 95% ice cold EtOH was added dropwise while vortexing. Cells were fixed in this final 70% Et-OH solution for at least 30 minutes or overnight. The Et-OH/cell suspension was then diluted with 12 ml of PBS (for a total volume of 15 ml) and centrifuge at 2000-2200 rpm for 10 min. Cells were then washed once more with 15 ml PBS and then resuspended in 0.5-2.0 ml of DAPI stain solution (0.1% TritonX 100 and 10 ug/ml). After 30 min of incubation on ice cells were analyzed by flow cytometry, measuring the fluorescence emission at 461 nm.

Apoptosis analysis by flow cytometry

BT-549 and MDA-MB231 cells were transfected with siRNA (control or against *LRRC4B*) and MCF-7 cells were transfected with *LRRC4B* overexpressing plasmid or control plasmid. Eight hours after transfection medium was changed and doxorubicin added according to the respective

IC50 for each cell line (6, 7). Cells were collected 60 hours post siRNA transfection or *LRRC4B* overexpression and 48 hours post treatment with doxorubicin respectively, stained with annexin V (Annexin V-FITC conjugate; Invitrogen, CO; #V13242) and propidium iodide (PI; Invitrogen, CO; #V13242), and analyzed by flow cytometry using the BD FACS Canto II cytometer (BD Biosciences, USA). Briefly, cells were harvested after incubation period and washed twice by centrifugation (1,200 g, 5 min) in cold phosphate-buffered saline (DPBS; Gibco, CO; #14040133). After washing, cells were resuspended in 0.15 ml AnnV binding buffer 1X (ABB 5X, Invitrogen, CO; #V13242; 50 mM HEPES, 700 mM NaCl, and 12.5 mM $CaCl_2$ at pH 7.4) containing fluorochrome-conjugated AnnV and PI (PI to a final concentration of 1 ug/ml) and incubated in darkness at room temperature for 15 min. As soon as possible cells were analyzed by flow cytometry, measuring the fluorescence emission at 530 nm and >575 nm.

SUPPLEMENTARY TABLE LEGENDS

Supplementary Table S1: Tables of all significant APSiC hits in the pan-cancer analysis

Supplementary Table S2: Significant genetic drivers identified by APSiC in the pan-cancer analysis of DRIVE

Supplementary Table S3: Tables of all significant APSiC hits in the mutation-level analysis

Supplementary Table S4: Tables of all significant APSiC hits in the cancer type-specific analysis

Supplementary Table S5: Clustering of the 500 most variable *P*-values (in terms of median absolute deviation) for effectors using the APSiC algorithm for the 26 cancer types in the DRIVE perturbation screen.

Supplementary Table S6: CRISPR validation for significant APSiC (DRIVE) hits from pan-cancer and cancer type analyses

REFERENCES

1. Wilkerson,M.D. and Hayes,D.N. (2010) ConsensusClusterPlus: a class discovery tool with confidence assessments and item tracking. *Bioinformatics*, **26**, 1572–1573.

2. Gaujoux,R. and Seoighe,C. (2010) A flexible R package for nonnegative matrix factorization. *BMC Bioinformatics*, **11**, 367.

3. Xu,T., Le,T.D., Liu,L., Su,N., Wang,R., Sun,B., Colaprico,A., Bontempi,G. and Li,J. (2017) CancerSubtypes: an R/Bioconductor package for molecular cancer subtype identification, validation and visualization. *Bioinformatics*, **33**, 3131–3133.

4. Yu,G., Wang,L.-G., Han,Y. and He,Q.-Y. (2012) clusterProfiler: an R Package for Comparing Biological Themes Among Gene Clusters. *OMICS: A Journal of Integrative Biology*, **16**, 284–287.

5. Ng,C.K.Y., Martelotto,L.G., Gauthier,A., Wen,H.-C., Piscuoglio,S., Lim,R.S., Cowell,C.F., Wilkerson,P.M., Wai,P., Rodrigues,D.N., *et al.* (2015) Intra-tumor genetic heterogeneity and alternative driver genetic alterations in breast cancers with heterogeneous HER2 gene amplification. *Genome Biol.*, **16**, 107.

6. Pilco-Ferreto,N. and Calaf,G.M. (2016) Influence of doxorubicin on apoptosis and oxidative stress in breast cancer cell lines. *Int. J. Oncol.*, **49**, 753–762.

7. Inao,T., Iida,Y., Moritani,T., Okimoto,T., Tanino,R., Kotani,H. and Harada,M. (2018) Bcl-2 inhibition sensitizes triple-negative human breast cancer cells to doxorubicin. *Oncotarget*, **9**, 25545–25556.
