# Supplementary material for: Systematic identification of novel cancer genes through analysis of deep shRNA perturbation screens
Source: Nucleic Acids Res. 2021 Jul 27;49(15):8488–504. doi: 10.1093/nar/gkab627 (PMC8421231; doi:10.1093/nar/gkab627)
Supplement: gkab627_Supplemental_Files [file gkab627_supplemental_files.zip › Montazeri et al. Supplementary Table S2.docx]

**Supplementary Table S2:** Significant genetic drivers identified by APSiC in the pan-cancer analysis of DRIVE

| **Gene** | **Predicted function** | **Known function** | **Cancer type** | **Mechanisms of action** |
| --- | --- | --- | --- | --- |
| *TP53* | Missense/ Non-missense mutational cancer gene | Oncogene/ Tumor suppressor | Leukemia, bladder cancer, breast cancer, melanoma, squamous cell carcinoma, liver cancer, kidney cancer, and others [(1)](https://paperpile.com/c/sM3jeU/MvA7o) | DNA repair, cell-cycle arrest, apoptosis, senescence, autophagy, angiogenesis [(1, 2)](https://paperpile.com/c/sM3jeU/56nBk+MvA7o) |
| *KRAS* | Missense mutational/ amplified cancer gene | Oncogene | Gastric cancer, squamous cell carcinoma, hepatic angiosarcoma, thyroid cancer, and others [(3)](https://paperpile.com/c/sM3jeU/NLLkW) | MAPK and PI3K-AKT pathways, cell proliferation and survival, metabolism [(3, 4)](https://paperpile.com/c/sM3jeU/NLLkW+xqwMe) |
| *BRAF* | Missense mutational/ amplified cancer gene | Oncogene | Colorectal cancer [(5)](https://paperpile.com/c/sM3jeU/UdLuR), thyroid cancer, melanoma, and others [(6)](https://paperpile.com/c/sM3jeU/uQatu) | MAPK pathway, cell proliferation and survival, differentiation [(5, 6)](https://paperpile.com/c/sM3jeU/uQatu+UdLuR) |
| *NRAS* | Missense mutational cancer gene | Oncogene | Gastric cancer, melanoma, angiosarcoma [(3)](https://paperpile.com/c/sM3jeU/NLLkW) | MAPK and PI3K-AKT pathways, cell proliferation and survival, metabolism, autophagy [(3, 4)](https://paperpile.com/c/sM3jeU/NLLkW+xqwMe) |
| *CTNNB1* | Missense mutational cancer gene | Oncogene | Gastric cancer, liver cancer[(7)](https://paperpile.com/c/sM3jeU/HyJvE), kidney cancer, colorectal cancer [(8)](https://paperpile.com/c/sM3jeU/UAyOn) | WNT pathway, cell proliferation and survival, epithelial-mesenchymal transition [(7, 9)](https://paperpile.com/c/sM3jeU/H3rsR+HyJvE) |
| *PIK3CA* | Missense mutational cancer gene | Oncogene | Ovarian cancer, breast cancer, liver cancer, and others [(10)](https://paperpile.com/c/sM3jeU/eQgZI) | PI3K-AKT pathway, cell proliferation and survival, metabolism, autophagy[(10, 11)](https://paperpile.com/c/sM3jeU/eQgZI+QHFW9) |
| *DDX27* | Missense mutational cancer gene | Oncogene | Colorectal cancer[(12)](https://paperpile.com/c/sM3jeU/drVLO), gastric cancer[(13)](https://paperpile.com/c/sM3jeU/kh13G) | NF-кB pathway, cell proliferation, migration, metastasis[(12)](https://paperpile.com/c/sM3jeU/drVLO), ribosomal RNA maturation [(14)](https://paperpile.com/c/sM3jeU/idWH5) |
| *MDM2* | Missense mutational cancer gene | Oncogene | Liposarcoma, breast cancer, glioblastoma, osteosarcoma, colorectal cancer, oesophageal cancer, melanoma [(15)](https://paperpile.com/c/sM3jeU/ShlRN), prostate cancer[(16)](https://paperpile.com/c/sM3jeU/bXlYi), HCC [(17)](https://paperpile.com/c/sM3jeU/ycnBi), | p53 regulation [(15)](https://paperpile.com/c/sM3jeU/ShlRN), mitochondrial respiration [(18)](https://paperpile.com/c/sM3jeU/V7so8), hormone receptors [(16)](https://paperpile.com/c/sM3jeU/bXlYi), serine metabolism and redox homeostasis [(19)](https://paperpile.com/c/sM3jeU/sFRm4) |
| *RNF40* | Missense mutational cancer gene | Oncogene/ Tumor suppressor | Leukemia[(20)](https://paperpile.com/c/sM3jeU/DIlTs), Colorectal cancer[(21)](https://paperpile.com/c/sM3jeU/56HxK), breast cancer [(22, 23)](https://paperpile.com/c/sM3jeU/oHOwG+1LrdR) | Histone ubiquitylation [(20, 24)](https://paperpile.com/c/sM3jeU/DIlTs+NGizy), NF-κB signalling and inflammation [(21)](https://paperpile.com/c/sM3jeU/56HxK), spindle assembly[(22)](https://paperpile.com/c/sM3jeU/oHOwG) |
| *MMS22L* | Missense/ Non-missense mutational cancer gene | Putative oncogene | Lung cancer [(25)](https://paperpile.com/c/sM3jeU/ynhDU), esophageal cancer [(25)](https://paperpile.com/c/sM3jeU/ynhDU), ovarian cancer [(26, 27)](https://paperpile.com/c/sM3jeU/czK32+LNT85) | DNA replication [(28)](https://paperpile.com/c/sM3jeU/jhkgp), genome stability [(28)](https://paperpile.com/c/sM3jeU/jhkgp), DNA repair [(26, 29)](https://paperpile.com/c/sM3jeU/czK32+F6c3Z), NF-кB pathway[(25)](https://paperpile.com/c/sM3jeU/ynhDU) |
| *CCNK* | Missense mutational cancer gene | Putative oncogene | Leukemia [(30)](https://paperpile.com/c/sM3jeU/6EIGM), ovarian carcinoma [(31)](https://paperpile.com/c/sM3jeU/eDMUH), prostate cancer [(32)](https://paperpile.com/c/sM3jeU/AOPX1) | DNA damage response [(30)](https://paperpile.com/c/sM3jeU/6EIGM), cell proliferation and cell cycle [(33)](https://paperpile.com/c/sM3jeU/W4QtJ), |
| *LRP1B* | Missense mutational cancer gene | Putative  tumor suppressor | Melanoma [(34)](https://paperpile.com/c/sM3jeU/TmZeB), lung cancer [(34)](https://paperpile.com/c/sM3jeU/TmZeB), ovarian cancer [(35)](https://paperpile.com/c/sM3jeU/jqK9k) | Tumor microenvironment and checkpoint blockade [(34, 36)](https://paperpile.com/c/sM3jeU/TmZeB+8aTv1), chemotherapy resistance [(35)](https://paperpile.com/c/sM3jeU/jqK9k) |
| *APC* | Non-missense mutational cancer gene | Tumor suppressor | Colorectal cancer[(37, 38)](https://paperpile.com/c/sM3jeU/eCTOZ+LcLe1), FAP [(39, 40)](https://paperpile.com/c/sM3jeU/2s4mb+9iskR) | Wnt/beta-catenin pathway, cell cycle [(39)](https://paperpile.com/c/sM3jeU/2s4mb), migration [(41)](https://paperpile.com/c/sM3jeU/4BdN8), chromosomal instability [(42, 43)](https://paperpile.com/c/sM3jeU/d3rT8+RLoxd) |
| *TTK* | Non-missense mutational cancer gene | Oncogene | Breast cancer [(44)](https://paperpile.com/c/sM3jeU/N9HYY), pancreatic cancer [(45)](https://paperpile.com/c/sM3jeU/AXfGT) lung cancer [(46)](https://paperpile.com/c/sM3jeU/inHII)and others [(47)](https://paperpile.com/c/sM3jeU/QtxXs) | Cell-cycle checkpoints, genome stability, DNA damage repair [(48)](https://paperpile.com/c/sM3jeU/jeeXY), p53 signalling [(49)](https://paperpile.com/c/sM3jeU/FLwI1) |
| *PTEN* | Non-missense mutational cancer gene | Tumor suppressor | Prostate cancer [(50)](https://paperpile.com/c/sM3jeU/DCFXU), PTEN hamartoma tumour syndrome (PHTS), tumours of the central nervous system, prostate, lung, pancreas, liver and adrenal glands, melanoma, leukaemia and lymphoma [(51)](https://paperpile.com/c/sM3jeU/n7tU9) | [PI3K–AKT–mTOR pathway](https://www.nature.com/articles/s41580-018-0015-0#Glos3) [(52)](https://paperpile.com/c/sM3jeU/V6bqf), cell motility and polarity, cell metabolism, genome maintenance, senescence, stemness [(52)](https://paperpile.com/c/sM3jeU/V6bqf) |
| *GEMIN5* | Non-missense mutational cancer gene | Putative tumor suppressor | Melanoma[(53)](https://paperpile.com/c/sM3jeU/DxIXB) | Alternative mRNA splicing[(54)](https://paperpile.com/c/sM3jeU/kVl1Y), tumor cell motility[(53)](https://paperpile.com/c/sM3jeU/DxIXB), MAPK signalling [(55)](https://paperpile.com/c/sM3jeU/xRMfA) |
| *USP7* | Non-missense mutational cancer gene | Putative oncogene | Colorectal cancer[(56)](https://paperpile.com/c/sM3jeU/kKFRX)**,** Leukemia[(57)](https://paperpile.com/c/sM3jeU/rP715), breast cancer[(58)](https://paperpile.com/c/sM3jeU/zaG0W), hepatocellular carcinoma [(59, 60)](https://paperpile.com/c/sM3jeU/NvDNW+zo2Pi) | WNT pathway[(56)](https://paperpile.com/c/sM3jeU/kKFRX), Notch signalling[(57)](https://paperpile.com/c/sM3jeU/rP715), ubiquitination[(61)](https://paperpile.com/c/sM3jeU/WbgRh), epigenetic regulation[(58)](https://paperpile.com/c/sM3jeU/zaG0W), p53 signalling[(62)](https://paperpile.com/c/sM3jeU/KzqQl), Hippo pathway [(59)](https://paperpile.com/c/sM3jeU/NvDNW) |
| *NOL11* | Non-missense mutational cancer gene | NA | NA | Nucleolar integrity[(63)](https://paperpile.com/c/sM3jeU/uEEIu), chromosome segregation[(64)](https://paperpile.com/c/sM3jeU/pazch), pre-rRNA transcription and processing[(65)](https://paperpile.com/c/sM3jeU/QqpLB) |
| *CDK4* | Amplified cancer gene | Oncogene | Non-small cell lung cancer[(66, 67)](https://paperpile.com/c/sM3jeU/fEXQk+aOLWM), cervical cancer[(66)](https://paperpile.com/c/sM3jeU/fEXQk), melanoma[(66, 68)](https://paperpile.com/c/sM3jeU/fEXQk+owwlj),  liposarcoma[(69)](https://paperpile.com/c/sM3jeU/VTdGy) and others | Cell-cycle check-point regulation, metabolism (anaerobic glycolysis)[(70)](https://paperpile.com/c/sM3jeU/vCej), epithelial-mesenchymal transition [(71)](https://paperpile.com/c/sM3jeU/4wxWA) |
| *IL6* | Amplified cancer gene | Oncogene | Prostate cancer[(72, 73)](https://paperpile.com/c/sM3jeU/YfLRh+kVfAw), breast cancer[(74)](https://paperpile.com/c/sM3jeU/sHFis), head and neck squamous cell carcinoma[(75)](https://paperpile.com/c/sM3jeU/fBCJz), liver cancer[(72)](https://paperpile.com/c/sM3jeU/YfLRh) | STAT3 activation [(76)](https://paperpile.com/c/sM3jeU/Pwduz), insulin-growth factor signaling [(77)](https://paperpile.com/c/sM3jeU/bHPEd), inflammation [(78)](https://paperpile.com/c/sM3jeU/B7VTV) |
| *TLK2* | Amplified cancer gene | Oncogene | Breast cancer[(79, 80)](https://paperpile.com/c/sM3jeU/d8H69+ViAe6), glioblastoma[(81)](https://paperpile.com/c/sM3jeU/4wZM4) | Genomic instability[(79)](https://paperpile.com/c/sM3jeU/d8H69), src signalling pathway[(81)](https://paperpile.com/c/sM3jeU/4wZM4) |
| *MITF* | Amplified cancer gene | Oncogene | Melanoma[(82)](https://paperpile.com/c/sM3jeU/ebSnc) | DNA replication, mitosis and genome stability [(82)](https://paperpile.com/c/sM3jeU/ebSnc), Wnt signaling [(83)](https://paperpile.com/c/sM3jeU/uhTn1) |

**Additional references**

[1. Giacomelli,A.O., Yang,X., Lintner,R.E., McFarland,J.M., Duby,M., Kim,J., Howard,T.P., Takeda,D.Y., Ly,S.H., Kim,E., *et al.* (2018) Mutational processes shape the landscape of TP53 mutations in human cancer. *Nat. Genet.*, **50**, 1381–1387.](http://paperpile.com/b/sM3jeU/MvA7o)

[2. Bykov,V.J.N., Eriksson,S.E., Bianchi,J. and Wiman,K.G. (2018) Targeting mutant p53 for efficient cancer therapy. *Nat. Rev. Cancer*, **18**, 89–102.](http://paperpile.com/b/sM3jeU/56nBk)

[3. Li,S., Balmain,A. and Counter,C.M. (2018) A model for RAS mutation patterns in cancers: finding the sweet spot. *Nature Reviews Cancer*, **18**, 767–777.](http://paperpile.com/b/sM3jeU/NLLkW)

[4. Roberts,P.J. and Der,C.J. (2007) Targeting the Raf-MEK-ERK mitogen-activated protein kinase cascade for the treatment of cancer. *Oncogene*, **26**, 3291–3310.](http://paperpile.com/b/sM3jeU/xqwMe)

[5. Sanz-Garcia,E., Argiles,G., Elez,E. and Tabernero,J. (2017) BRAF mutant colorectal cancer: prognosis, treatment, and new perspectives. *Ann. Oncol.*, **28**, 2648–2657.](http://paperpile.com/b/sM3jeU/UdLuR)

[6. Dankner,M., Rose,A.A.N., Rajkumar,S., Siegel,P.M. and Watson,I.R. (2018) Classifying BRAF alterations in cancer: new rational therapeutic strategies for actionable mutations. *Oncogene*, **37**, 3183–3199.](http://paperpile.com/b/sM3jeU/uQatu)

[7. Zucman-Rossi,J., Villanueva,A., Nault,J.-C. and Llovet,J.M. (2015) Genetic Landscape and Biomarkers of Hepatocellular Carcinoma. *Gastroenterology*, **149**, 1226–1239.e4.](http://paperpile.com/b/sM3jeU/HyJvE)

[8. Wu,H., Lu,X.-X., Wang,J.-R., Yang,T.-Y., Li,X.-M., He,X.-S., Li,Y., Ye,W.-L., Wu,Y., Gan,W.-J., *et al.* (2019) TRAF6 inhibits colorectal cancer metastasis through regulating selective autophagic CTNNB1/β-catenin degradation and is targeted for GSK3B/GSK3β-mediated phosphorylation and degradation. *Autophagy*, **15**, 1506–1522.](http://paperpile.com/b/sM3jeU/UAyOn)

[9. Monga,S.P. (2015) β-Catenin Signaling and Roles in Liver Homeostasis, Injury, and Tumorigenesis. *Gastroenterology*, **148**, 1294–1310.](http://paperpile.com/b/sM3jeU/H3rsR)

[10. Karakas,B., Bachman,K.E. and Park,B.H. (2006) Mutation of the PIK3CA oncogene in human cancers. *British Journal of Cancer*, **94**, 455–459.](http://paperpile.com/b/sM3jeU/eQgZI)

[11. Madsen,R.R., Knox,R.G., Pearce,W., Lopez,S., Mahler-Araujo,B., McGranahan,N., Vanhaesebroeck,B. and Semple,R.K. Oncogenic PIK3CA promotes cellular stemness in an allele dose-dependent manner.](http://paperpile.com/b/sM3jeU/QHFW9) [10.1101/495093](http://dx.doi.org/10.1101/495093)[.](http://paperpile.com/b/sM3jeU/QHFW9)

[12. Tang,J., Chen,H., Wong,C.-C., Liu,D., Li,T., Wang,X., Ji,J., Sung,J.J., Fang,J.-Y. and Yu,J. (2018) DEAD-box helicase 27 promotes colorectal cancer growth and metastasis and predicts poor survival in CRC patients. *Oncogene*, **37**, 3006–3021.](http://paperpile.com/b/sM3jeU/drVLO)

[13. Tsukamoto,Y., Fumoto,S., Noguchi,T., Yanagihara,K., Hirashita,Y., Nakada,C., Hijiya,N., Uchida,T., Matsuura,K., Hamanaka,R., *et al.* (2015) Expression of DDX27 contributes to colony-forming ability of gastric cancer cells and correlates with poor prognosis in gastric cancer. *Am. J. Cancer Res.*, **5**, 2998–3014.](http://paperpile.com/b/sM3jeU/kh13G)

[14. Bennett,A.H., O’Donohue,M.-F., Gundry,S.R., Chan,A.T., Widrick,J., Draper,I., Chakraborty,A., Zhou,Y., Zon,L.I., Gleizes,P.-E., *et al.* (2018) RNA helicase, DDX27 regulates skeletal muscle growth and regeneration by modulation of translational processes. *PLoS Genet.*, **14**, e1007226.](http://paperpile.com/b/sM3jeU/idWH5)

[15. Wade,M., Li,Y.-C. and Wahl,G.M. (2013) MDM2, MDMX and p53 in oncogenesis and cancer therapy. *Nat. Rev. Cancer*, **13**, 83–96.](http://paperpile.com/b/sM3jeU/ShlRN)

[16. Vummidi Giridhar,P., Williams,K., VonHandorf,A.P., Deford,P.L. and Kasper,S. (2019) Constant Degradation of the Androgen Receptor by MDM2 Conserves Prostate Cancer Stem Cell Integrity. *Cancer Res.*, **79**, 1124–1137.](http://paperpile.com/b/sM3jeU/bXlYi)

[17. Wang,W., Cheng,J.-W., Qin,J.-J., Hu,B., Li,X., Nijampatnam,B., Velu,S.E., Fan,J., Yang,X.-R. and Zhang,R. (2019) MDM2-NFAT1 dual inhibitor, MA242: Effective against hepatocellular carcinoma, independent of p53. *Cancer Lett.*, **459**, 156–167.](http://paperpile.com/b/sM3jeU/ycnBi)

[18. Arena,G., Cissé,M.Y., Pyrdziak,S., Chatre,L., Riscal,R., Fuentes,M., Arnold,J.J., Kastner,M., Gayte,L., Bertrand-Gaday,C., *et al.* (2018) Mitochondrial MDM2 Regulates Respiratory Complex I Activity Independently of p53. *Mol. Cell*, **69**, 594–609.e8.](http://paperpile.com/b/sM3jeU/V7so8)

[19. Riscal,R., Schrepfer,E., Arena,G., Cissé,M.Y., Bellvert,F., Heuillet,M., Rambow,F., Bonneil,E., Sabourdy,F., Vincent,C., *et al.* (2016) Chromatin-Bound MDM2 Regulates Serine Metabolism and Redox Homeostasis Independently of p53. *Mol. Cell*, **62**, 890–902.](http://paperpile.com/b/sM3jeU/sFRm4)

[20. Castro,P.G., Garrido Castro,P., van Roon,E.H.J., Pinhanços,S.S., Trentin,L., Schneider,P., Kerstjens,M., te Kronnie,G., Heidenreich,O., Pieters,R., *et al.* (2018) The HDAC inhibitor panobinostat (LBH589) exerts in vivo anti-leukaemic activity against MLL-rearranged acute lymphoblastic leukaemia and involves the RNF20/RNF40/WAC-H2B ubiquitination axis. *Leukemia*, **32**, 323–331.](http://paperpile.com/b/sM3jeU/DIlTs)

[21. Tarcic,O., Pateras,I.S., Cooks,T., Shema,E., Kanterman,J., Ashkenazi,H., Boocholez,H., Hubert,A., Rotkopf,R., Baniyash,M., *et al.* (2016) RNF20 Links Histone H2B Ubiquitylation with Inflammation and Inflammation-Associated Cancer. *Cell Rep.*, **14**, 1462–1476.](http://paperpile.com/b/sM3jeU/56HxK)

[22. Duan,Y., Huo,D., Gao,J., Wu,H., Ye,Z., Liu,Z., Zhang,K., Shan,L., Zhou,X., Wang,Y., *et al.* (2016) Corrigendum: Ubiquitin ligase RNF20/40 facilitates spindle assembly and promotes breast carcinogenesis through stabilizing motor protein Eg5. *Nat. Commun.*, **7**, 13462.](http://paperpile.com/b/sM3jeU/oHOwG)

[23. Prenzel,T., Begus-Nahrmann,Y., Kramer,F., Hennion,M., Hsu,C., Gorsler,T., Hintermair,C., Eick,D., Kremmer,E., Simons,M., *et al.* (2011) Estrogen-dependent gene transcription in human breast cancer cells relies upon proteasome-dependent monoubiquitination of histone H2B. *Cancer Res.*, **71**, 5739–5753.](http://paperpile.com/b/sM3jeU/1LrdR)

[24. Segala,G., Bennesch,M.A., Pandey,D.P., Hulo,N. and Picard,D. (2016) Monoubiquitination of Histone H2B Blocks Eviction of Histone Variant H2A.Z from Inducible Enhancers. *Molecular Cell*, **64**, 334–346.](http://paperpile.com/b/sM3jeU/NGizy)

[25. Nguyen,M.-H., Ueda,K., Nakamura,Y. and Daigo,Y. (2012) Identification of a novel oncogene, MMS22L, involved in lung and esophageal carcinogenesis. *Int. J. Oncol.*, **41**, 1285–1296.](http://paperpile.com/b/sM3jeU/ynhDU)

[26. Piwko,W., Mlejnkova,L.J., Mutreja,K., Ranjha,L., Stafa,D., Smirnov,A., Brodersen,M.M.L., Zellweger,R., Sturzenegger,A., Janscak,P., *et al.* (2016) The MMS22L–TONSL heterodimer directly promotes RAD51‐dependent recombination upon replication stress. *The EMBO Journal*, **35**, 2584–2601.](http://paperpile.com/b/sM3jeU/czK32)

[27. Forbes,S.A., Beare,D., Gunasekaran,P., Leung,K., Bindal,N., Boutselakis,H., Ding,M., Bamford,S., Cole,C., Ward,S., *et al.* (2015) COSMIC: exploring the world’s knowledge of somatic mutations in human cancer. *Nucleic Acids Research*, **43**, D805–D811.](http://paperpile.com/b/sM3jeU/LNT85)

[28. Piwko,W., Olma,M.H., Held,M., Bianco,J.N., Pedrioli,P.G.A., Hofmann,K., Pasero,P., Gerlich,D.W. and Peter,M. (2010) RNAi-based screening identifies the Mms22L–Nfkbil2 complex as a novel regulator of DNA replication in human cells. *The EMBO Journal*, **29**, 4210–4222.](http://paperpile.com/b/sM3jeU/jhkgp)

[29. Duro,E., Lundin,C., Ask,K., Sanchez-Pulido,L., MacArtney,T.J., Toth,R., Ponting,C.P., Groth,A., Helleday,T. and Rouse,J. (2010) Identification of the MMS22L-TONSL complex that promotes homologous recombination. *Mol. Cell*, **40**, 632–644.](http://paperpile.com/b/sM3jeU/F6c3Z)

[30. Hoshii,T., Cifani,P., Feng,Z., Huang,C.-H., Koche,R., Chen,C.-W., Delaney,C.D., Lowe,S.W., Kentsis,A. and Armstrong,S.A. (2018) A Non-catalytic Function of SETD1A Regulates Cyclin K and the DNA Damage Response. *Cell*, **172**, 1007–1021.e17.](http://paperpile.com/b/sM3jeU/6EIGM)

[31. Ekumi,K.M., Paculova,H., Lenasi,T., Pospichalova,V., Bösken,C.A., Rybarikova,J., Bryja,V., Geyer,M., Blazek,D. and Barboric,M. (2015) Ovarian carcinoma CDK12 mutations misregulate expression of DNA repair genes via deficient formation and function of the Cdk12/CycK complex. *Nucleic Acids Res.*, **43**, 2575–2589.](http://paperpile.com/b/sM3jeU/eDMUH)

[32. Schecher,S., Walter,B., Falkenstein,M., Macher-Goeppinger,S., Stenzel,P., Krümpelmann,K., Hadaschik,B., Perner,S., Kristiansen,G., Duensing,S., *et al.* (2017) Cyclin K dependent regulation of Aurora B affects apoptosis and proliferation by induction of mitotic catastrophe in prostate cancer. *Int. J. Cancer*, **141**, 1643–1653.](http://paperpile.com/b/sM3jeU/AOPX1)

[33. Lei,T., Zhang,P., Zhang,X., Xiao,X., Zhang,J., Qiu,T., Dai,Q., Zhang,Y., Min,L., Li,Q., *et al.* (2018) Cyclin K regulates prereplicative complex assembly to promote mammalian cell proliferation. *Nat. Commun.*, **9**, 1876.](http://paperpile.com/b/sM3jeU/W4QtJ)

[34. Chen,H., Chong,W., Wu,Q., Yao,Y., Mao,M. and Wang,X. (2019) Association of LRP1B Mutation With Tumor Mutation Burden and Outcomes in Melanoma and Non-small Cell Lung Cancer Patients Treated With Immune Check-Point Blockades. *Frontiers in Immunology*, **10**.](http://paperpile.com/b/sM3jeU/TmZeB)

[35. Cowin,P.A., George,J., Fereday,S., Loehrer,E., Van Loo,P., Cullinane,C., Etemadmoghadam,D., Ftouni,S., Galletta,L., Anglesio,M.S., *et al.* (2012) LRP1B deletion in high-grade serous ovarian cancers is associated with acquired chemotherapy resistance to liposomal doxorubicin. *Cancer Res.*, **72**, 4060–4073.](http://paperpile.com/b/sM3jeU/jqK9k)

[36. Prazeres,H., Torres,J., Rodrigues,F., Pinto,M., Pastoriza,M.C., Gomes,D., Cameselle-Teijeiro,J., Vidal,A., Martins,T.C., Sobrinho-Simões,M., *et al.* (2017) Chromosomal, epigenetic and microRNA-mediated inactivation of LRP1B, a modulator of the extracellular environment of thyroid cancer cells. *Oncogene*, **36**, 146.](http://paperpile.com/b/sM3jeU/8aTv1)

[37. Rowan,A.J., Lamlum,H., Ilyas,M., Wheeler,J., Straub,J., Papadopoulou,A., Bicknell,D., Bodmer,W.F. and Tomlinson,I.P. (2000) APC mutations in sporadic colorectal tumors: A mutational ‘hotspot’ and interdependence of the ‘two hits’. *Proc. Natl. Acad. Sci. U. S. A.*, **97**, 3352–3357.](http://paperpile.com/b/sM3jeU/eCTOZ)

[38. Powell,S.M., Zilz,N., Beazer-Barclay,Y., Bryan,T.M., Hamilton,S.R., Thibodeau,S.N., Vogelstein,B. and Kinzler,K.W. (1992) APC mutations occur early during colorectal tumorigenesis. *Nature*, **359**, 235–237.](http://paperpile.com/b/sM3jeU/LcLe1)

[39. Zhang,L. and Shay,J.W. (2017) Multiple Roles of APC and its Therapeutic Implications in Colorectal Cancer. *J. Natl. Cancer Inst.*, **109**.](http://paperpile.com/b/sM3jeU/2s4mb)

[40. Kinzler,K.W. and Vogelstein,B. (1996) Lessons from hereditary colorectal cancer. *Cell*, **87**, 159–170.](http://paperpile.com/b/sM3jeU/9iskR)

[41. Kawasaki,Y., Sato,R. and Akiyama,T. (2003) Mutated APC and Asef are involved in the migration of colorectal tumour cells. *Nat. Cell Biol.*, **5**, 211–215.](http://paperpile.com/b/sM3jeU/4BdN8)

[42. Sansregret,L., Patterson,J.O., Dewhurst,S., López-García,C., Koch,A., McGranahan,N., Chao,W.C.H., Barry,D.J., Rowan,A., Instrell,R., *et al.* (2017) APC/C Dysfunction Limits Excessive Cancer Chromosomal Instability. *Cancer Discov.*, **7**, 218–233.](http://paperpile.com/b/sM3jeU/d3rT8)

[43. Kaplan,K.B., Burds,A.A., Swedlow,J.R., Bekir,S.S., Sorger,P.K. and Näthke,I.S. (2001) A role for the Adenomatous Polyposis Coli protein in chromosome segregation. *Nat. Cell Biol.*, **3**, 429–432.](http://paperpile.com/b/sM3jeU/RLoxd)

[44. Thu,K.L., Silvester,J., Elliott,M.J., Ba-Alawi,W., Duncan,M.H., Elia,A.C., Mer,A.S., Smirnov,P., Safikhani,Z., Haibe-Kains,B., *et al.* (2018) Disruption of the anaphase-promoting complex confers resistance to TTK inhibitors in triple-negative breast cancer. *Proc. Natl. Acad. Sci. U. S. A.*, **115**, E1570–E1577.](http://paperpile.com/b/sM3jeU/N9HYY)

[45. Slee,R.B., Grimes,B.R., Bansal,R., Gore,J., Blackburn,C., Brown,L., Gasaway,R., Jeong,J., Victorino,J., March,K.L., *et al.* (2014) Selective inhibition of pancreatic ductal adenocarcinoma cell growth by the mitotic MPS1 kinase inhibitor NMS-P715. *Mol. Cancer Ther.*, **13**, 307–315.](http://paperpile.com/b/sM3jeU/AXfGT)

[46. Chen,X., Yu,C., Gao,J., Zhu,H., Cui,B., Zhang,T., Zhou,Y., Liu,Q., He,H., Xiao,R., *et al.* (2018) A novel USP9X substrate TTK contributes to tumorigenesis in non-small-cell lung cancer. *Theranostics*, **8**, 2348–2360.](http://paperpile.com/b/sM3jeU/inHII)

[47. Carter,S.L., Eklund,A.C., Kohane,I.S., Harris,L.N. and Szallasi,Z. (2006) A signature of chromosomal instability inferred from gene expression profiles predicts clinical outcome in multiple human cancers. *Nat. Genet.*, **38**, 1043–1048.](http://paperpile.com/b/sM3jeU/QtxXs)

[48. Yu,Z.-C., Huang,Y.-F. and Shieh,S.-Y. (2016) Requirement for human Mps1/TTK in oxidative DNA damage repair and cell survival through MDM2 phosphorylation. *Nucleic Acids Res.*, **44**, 1133–1150.](http://paperpile.com/b/sM3jeU/jeeXY)

[49. Huang,Y.-F., Chang,M.D.-T. and Shieh,S.-Y. (2009) TTK/hMps1 mediates the p53-dependent postmitotic checkpoint by phosphorylating p53 at Thr18. *Mol. Cell. Biol.*, **29**, 2935–2944.](http://paperpile.com/b/sM3jeU/FLwI1)

[50. Jamaspishvili,T., Berman,D.M., Ross,A.E., Scher,H.I., De Marzo,A.M., Squire,J.A. and Lotan,T.L. (2018) Clinical implications of PTEN loss in prostate cancer. *Nat. Rev. Urol.*, **15**, 222–234.](http://paperpile.com/b/sM3jeU/DCFXU)

[51. Hollander,M.C., Blumenthal,G.M. and Dennis,P.A. (2011) PTEN loss in the continuum of common cancers, rare syndromes and mouse models. *Nat. Rev. Cancer*, **11**, 289–301.](http://paperpile.com/b/sM3jeU/n7tU9)

[52. Lee,Y.-R., Chen,M. and Pandolfi,P.P. (2018) The functions and regulation of the PTEN tumour suppressor: new modes and prospects. *Nature Reviews Molecular Cell Biology*, **19**, 547–562.](http://paperpile.com/b/sM3jeU/V6bqf)

[53. Lee,J.H., Horak,C.E., Khanna,C., Meng,Z., Yu,L.R., Veenstra,T.D. and Steeg,P.S. (2008) Alterations in Gemin5 expression contribute to alternative mRNA splicing patterns and tumor cell motility. *Cancer Res.*, **68**, 639–644.](http://paperpile.com/b/sM3jeU/DxIXB)

[54. Francisco-Velilla,R., Fernandez-Chamorro,J., Ramajo,J. and Martinez-Salas,E. (2016) The RNA-binding protein Gemin5 binds directly to the ribosome and regulates global translation. *Nucleic Acids Res.*, **44**, 8335–8351.](http://paperpile.com/b/sM3jeU/kVl1Y)

[55. Kim,E.K., Noh,K.T., Yoon,J.-H., Cho,J.-H., Yoon,K.W., Dreyfuss,G. and Choi,E.-J. (2007) Positive regulation of ASK1-mediated c-Jun NH2-terminal kinase signaling pathway by the WD-repeat protein Gemin5. *Cell Death & Differentiation*, **14**, 1518–1528.](http://paperpile.com/b/sM3jeU/xRMfA)

[56. Novellasdemunt,L., Foglizzo,V., Cuadrado,L., Antas,P., Kucharska,A., Encheva,V., Snijders,A.P. and Li,V.S.W. (2017) USP7 Is a Tumor-Specific WNT Activator for APC-Mutated Colorectal Cancer by Mediating β-Catenin Deubiquitination. *Cell Rep.*, **21**, 612–627.](http://paperpile.com/b/sM3jeU/kKFRX)

[57. Jin,Q., Martinez,C.A., Arcipowski,K.M., Zhu,Y., Gutierrez-Diaz,B.T., Wang,K.K., Johnson,M.R., Volk,A.G., Wang,F., Wu,J., *et al.* (2019) USP7 Cooperates with NOTCH1 to Drive the Oncogenic Transcriptional Program in T-Cell Leukemia. *Clin. Cancer Res.*, **25**, 222–239.](http://paperpile.com/b/sM3jeU/rP715)

[58. Wang,Q., Ma,S., Song,N., Li,X., Liu,L., Yang,S., Ding,X., Shan,L., Zhou,X., Su,D., *et al.* (2016) Stabilization of histone demethylase PHF8 by USP7 promotes breast carcinogenesis. *J. Clin. Invest.*, **126**, 2205–2220.](http://paperpile.com/b/sM3jeU/zaG0W)

[59. Sun,X., Ding,Y., Zhan,M., Li,Y., Gao,D., Wang,G., Gao,Y., Li,Y., Wu,S., Lu,L., *et al.* (2019) Usp7 regulates Hippo pathway through deubiquitinating the transcriptional coactivator Yorkie. *Nat. Commun.*, **10**, 411.](http://paperpile.com/b/sM3jeU/NvDNW)

[60. Zhang,H., Deng,T., Ge,S., Liu,Y., Bai,M., Zhu,K., Fan,Q., Li,J., Ning,T., Tian,F., *et al.* (2019) Exosome circRNA secreted from adipocytes promotes the growth of hepatocellular carcinoma by targeting deubiquitination-related USP7. *Oncogene*, **38**, 2844–2859.](http://paperpile.com/b/sM3jeU/zo2Pi)

[61. Kategaya,L., Di Lello,P., Rougé,L., Pastor,R., Clark,K.R., Drummond,J., Kleinheinz,T., Lin,E., Upton,J.-P., Prakash,S., *et al.* (2017) USP7 small-molecule inhibitors interfere with ubiquitin binding. *Nature*, **550**, 534–538.](http://paperpile.com/b/sM3jeU/WbgRh)

[62. Schauer,N.J., Liu,X., Magin,R.S., Doherty,L.M., Chan,W.C., Ficarro,S.B., Hu,W., Roberts,R.M., Iacob,R.E., Stolte,B., *et al.* (2020) Selective USP7 inhibition elicits cancer cell killing through a p53-dependent mechanism. *Sci. Rep.*, **10**, 5324.](http://paperpile.com/b/sM3jeU/KzqQl)

[63. Hayashi,Y., Fujimura,A., Kato,K., Udagawa,R., Hirota,T. and Kimura,K. (2018) Nucleolar integrity during interphase supports faithful Cdk1 activation and mitotic entry. *Sci Adv*, **4**, eaap7777.](http://paperpile.com/b/sM3jeU/uEEIu)

[64. Fujimura,A., Hayashi,Y., Kato,K., Kogure,Y., Kameyama,M., Shimamoto,H., Daitoku,H., Fukamizu,A., Hirota,T. and Kimura,K. (2020) Identification of a novel nucleolar protein complex required for mitotic chromosome segregation through centromeric accumulation of Aurora B. *Nucleic Acids Res.*, **48**, 6583–6596.](http://paperpile.com/b/sM3jeU/pazch)

[65. Freed,E.F., Prieto,J.-L., McCann,K.L., McStay,B. and Baserga,S.J. (2012) NOL11, implicated in the pathogenesis of North American Indian childhood cirrhosis, is required for pre-rRNA transcription and processing. *PLoS Genet.*, **8**, e1002892.](http://paperpile.com/b/sM3jeU/QqpLB)

[66. Cheung,T.H., Yu,M.M., Lo,K.W., Yim,S.F., Chung,T.K. and Wong,Y.F. (2001) Alteration of cyclin D1 and CDK4 gene in carcinoma of uterine cervix. *Cancer Lett.*, **166**, 199–206.](http://paperpile.com/b/sM3jeU/fEXQk)

[67. Jamal-Hanjani,M., Wilson,G.A., McGranahan,N., Birkbak,N.J., Watkins,T.B.K., Veeriah,S., Shafi,S., Johnson,D.H., Mitter,R., Rosenthal,R., *et al.* (2017) Tracking the Evolution of Non-Small-Cell Lung Cancer. *N. Engl. J. Med.*, **376**, 2109–2121.](http://paperpile.com/b/sM3jeU/aOLWM)

[68. Curtin,J.A., Fridlyand,J., Kageshita,T., Patel,H.N., Busam,K.J., Kutzner,H., Cho,K.-H., Aiba,S., Bröcker,E.-B., LeBoit,P.E., *et al.* (2005) Distinct sets of genetic alterations in melanoma. *N. Engl. J. Med.*, **353**, 2135–2147.](http://paperpile.com/b/sM3jeU/owwlj)

[69. Wong,D.D., Low,I.C., Peverall,J., Robbins,P.D., Spagnolo,D.V., Nairn,R., Carey-Smith,R.L. and Wood,D. (2016) MDM2/CDK4 gene amplification in large/deep-seated ‘lipomas’: incidence, predictors and clinical significance. *Pathology*, **48**, 203–209.](http://paperpile.com/b/sM3jeU/VTdGy)

[70. Lopez-Mejia,I.C., Lagarrigue,S., Giralt,A., Martinez-Carreres,L., Zanou,N., Denechaud,P.-D., Castillo-Armengol,J., Chavey,C., Orpinell,M., Delacuisine,B., *et al.* (2017) CDK4 Phosphorylates AMPKα2 to Inhibit Its Activity and Repress Fatty Acid Oxidation. *Mol. Cell*, **68**, 336–349.e6.](http://paperpile.com/b/sM3jeU/vCej)

[71. Liu,T., Yu,J., Deng,M., Yin,Y., Zhang,H., Luo,K., Qin,B., Li,Y., Wu,C., Ren,T., *et al.* (2017) CDK4/6-dependent activation of DUB3 regulates cancer metastasis through SNAIL1. *Nat. Commun.*, **8**, 13923.](http://paperpile.com/b/sM3jeU/4wxWA)

[72. He,G., Dhar,D., Nakagawa,H., Font-Burgada,J., Ogata,H., Jiang,Y., Shalapour,S., Seki,E., Yost,S.E., Jepsen,K., *et al.* (2013) Identification of liver cancer progenitors whose malignant progression depends on autocrine IL-6 signaling. *Cell*, **155**, 384–396.](http://paperpile.com/b/sM3jeU/YfLRh)

[73. Liu,G., Zhang,J., Frey,L., Gang,X., Wu,K., Liu,Q., Lilly,M. and Wu,J. (2017) Prostate-specific IL-6 transgene autonomously induce prostate neoplasm through amplifying inflammation in the prostate and peri-prostatic adipose tissue. *J. Hematol. Oncol.*, **10**, 14.](http://paperpile.com/b/sM3jeU/kVfAw)

[74. Hartman,Z.C., Poage,G.M., den Hollander,P., Tsimelzon,A., Hill,J., Panupinthu,N., Zhang,Y., Mazumdar,A., Hilsenbeck,S.G., Mills,G.B., *et al.* (2013) Growth of triple-negative breast cancer cells relies upon coordinate autocrine expression of the proinflammatory cytokines IL-6 and IL-8. *Cancer Res.*, **73**, 3470–3480.](http://paperpile.com/b/sM3jeU/sHFis)

[75. Yun,M.R., Choi,H.M., Kang,H.N., Lee,Y., Joo,H.-S., Kim,D.H., Kim,H.R., Hong,M.H., Yoon,S.O. and Cho,B.C. (2018) ERK-dependent IL-6 autocrine signaling mediates adaptive resistance to pan-PI3K inhibitor BKM120 in head and neck squamous cell carcinoma. *Oncogene*, **37**, 377–388.](http://paperpile.com/b/sM3jeU/fBCJz)

[76. Johnson,D.E., O’Keefe,R.A. and Grandis,J.R. (2018) Targeting the IL-6/JAK/STAT3 signalling axis in cancer. *Nature Reviews Clinical Oncology*, **15**, 234–248.](http://paperpile.com/b/sM3jeU/Pwduz)

[77. Chang,T.-S., Wu,Y.-C., Chi,C.-C., Su,W.-C., Chang,P.-J., Lee,K.-F., Tung,T.-H., Wang,J., Liu,J.-J., Tung,S.-Y., *et al.* (2015) Activation of IL6/IGFIR Confers Poor Prognosis of HBV-Related Hepatocellular Carcinoma through Induction of OCT4/NANOG Expression. *Clinical Cancer Research*, **21**, 201–210.](http://paperpile.com/b/sM3jeU/bHPEd)

[78. Hayashi,T., Fujita,K., Nojima,S., Hayashi,Y., Nakano,K., Ishizuya,Y., Wang,C., Yamamoto,Y., Kinouchi,T., Matsuzaki,K., *et al.* (2018) High-Fat Diet-Induced Inflammation Accelerates Prostate Cancer Growth via IL6 Signaling. *Clin. Cancer Res.*, **24**, 4309–4318.](http://paperpile.com/b/sM3jeU/B7VTV)

[79. Kim,J.-A., Anurag,M., Veeraraghavan,J., Schiff,R., Li,K. and Wang,X.-S. (2016) Amplification of TLK2 Induces Genomic Instability via Impairing the G2-M Checkpoint. *Mol. Cancer Res.*, **14**, 920–927.](http://paperpile.com/b/sM3jeU/d8H69)

[80. Kim,J.-A., Tan,Y., Wang,X., Cao,X., Veeraraghavan,J., Liang,Y., Edwards,D.P., Huang,S., Pan,X., Li,K., *et al.* (2016) Comprehensive functional analysis of the tousled-like kinase 2 frequently amplified in aggressive luminal breast cancers. *Nat. Commun.*, **7**, 12991.](http://paperpile.com/b/sM3jeU/ViAe6)

[81. Lin,M., Yao,Z., Zhao,N. and Zhang,C. (2019) TLK2 enhances aggressive phenotypes of glioblastoma cells through the activation of SRC signaling pathway. *Cancer Biol. Ther.*, **20**, 101–108.](http://paperpile.com/b/sM3jeU/4wZM4)

[82. Strub,T., Giuliano,S., Ye,T., Bonet,C., Keime,C., Kobi,D., Le Gras,S., Cormont,M., Ballotti,R., Bertolotto,C., *et al.* (2011) Essential role of microphthalmia transcription factor for DNA replication, mitosis and genomic stability in melanoma. *Oncogene*, **30**, 2319–2332.](http://paperpile.com/b/sM3jeU/ebSnc)

[83. Ploper,D. and De Robertis,E.M. (2015) The MITF family of transcription factors: Role in endolysosomal biogenesis, Wnt signaling, and oncogenesis. *Pharmacol. Res.*, **99**, 36–43.](http://paperpile.com/b/sM3jeU/uhTn1)
